# Prenatal Exposure to Chemical Mixtures and Metabolic Syndrome Risk in Children

**DOI:** 10.1001/jamanetworkopen.2024.12040

**Published:** 2024-05-23

**Authors:** Nuria Güil-Oumrait, Nikos Stratakis, Léa Maitre, Augusto Anguita-Ruiz, Jose Urquiza, Lorenzo Fabbri, Xavier Basagaña, Barbara Heude, Line Småstuen Haug, Amrit Kaur Sakhi, Nina Iszatt, Hector C. Keun, John Wright, Leda Chatzi, Marina Vafeiadi, Mariona Bustamante, Regina Grazuleviciene, Sandra Andrušaitytė, Rémy Slama, Rosemary McEachan, Maribel Casas, Martine Vrijheid

**Affiliations:** 1 Barcelona Institute for Global Health (ISGlobal), Barcelona, Spain; 2 Pompeu Fabra University, Barcelona, Spain; 3 Centro de Investigación Biomédica en Red de Epidemiología y Salud Pública (CIBERESP), Madrid, Spain; 4 Université Paris Cité and Université Sorbonne Paris Nord, National Institute of Health and Medical Research (INSERM), National Institute for Agriculture, Food and the Environment (INRAE), Center for Research in Epidemiology and StatisticS (CRESS), Paris, France; 5 Department of Food Safety, Norwegian Institute of Public Health, Oslo, Norway; 6 Division of Climate and Environmental Health, Norwegian Institute of Public Health, Oslo, Norway; 7 Cancer Metabolism & Systems Toxicology Group, Imperial College London, Hammersmith Hospital Campus, London, United Kingdom; 8 Bradford Institute for Health Research, Bradford Teaching Hospitals National Health Service Foundation Trust, Bradford, United Kingdom; 9 Department of Preventive Medicine, Keck School of Medicine, University of Southern California, Los Angeles; 10 Department of Social Medicine, University of Crete, Heraklion, Crete, Greece; 11 Department of Environmental Sciences, Vytautas Magnus University, Kaunas, Lithuania; 12 Department of Prevention and Treatment of Chronic Diseases, Institute for Advanced Biosciences (IAB; INSERM U1209, CNRS UMR 5309), Université Grenoble Alpes, Grenoble, France

## Abstract

**Importance:**

Prenatal exposure to ubiquitous endocrine-disrupting chemicals (EDCs) may increase the risk of metabolic syndrome (MetS) in children, but few studies have studied chemical mixtures or explored underlying protein and metabolic signatures.

**Objective:**

To investigate associations of prenatal exposure to EDC mixtures with MetS risk score in children and identify associated proteins and metabolites.

**Design, Setting, and Participants:**

This population-based, birth cohort study used data collected between April 1, 2003, and February 26, 2016, from the Human Early Life Exposome cohort based in France, Greece, Lithuania, Norway, Spain, and the UK. Eligible participants included mother-child pairs with measured prenatal EDC exposures and complete data on childhood MetS risk factors, proteins, and metabolites. Data were analyzed between October 2022 and July 2023.

**Exposures:**

Nine metals, 3 organochlorine pesticides, 5 polychlorinated biphenyls, 2 polybrominated diphenyl ethers (PBDEs), 5 perfluoroalkyl substances (PFAS), 10 phthalate metabolites, 3 phenols, 4 parabens, and 4 organophosphate pesticide metabolites measured in urine and blood samples collected during pregnancy.

**Main Outcomes and Measures:**

At 6 to 11 years of age, a composite MetS risk score was constructed using *z* scores of waist circumference, systolic and diastolic blood pressures, triglycerides, high-density lipoprotein cholesterol, and insulin levels. Childhood levels of 44 urinary metabolites, 177 serum metabolites, and 35 plasma proteins were quantified using targeted methods. Associations were assessed using bayesian weighted quantile sum regressions applied to mixtures for each chemical group.

**Results:**

The study included 1134 mothers (mean [SD] age at birth, 30.7 [4.9] years) and their children (mean [SD] age, 7.8 [1.5] years; 617 male children [54.4%] and 517 female children [45.6%]; mean [SD] MetS risk score, −0.1 [2.3]). MetS score increased per 1-quartile increase of the mixture for metals (β = 0.44; 95% credible interval [CrI], 0.30 to 0.59), organochlorine pesticides (β = 0.22; 95% CrI, 0.15 to 0.29), PBDEs (β = 0.17; 95% CrI, 0.06 to 0.27), and PFAS (β = 0.19; 95% CrI, 0.14 to 0.24). High-molecular weight phthalate mixtures (β = −0.07; 95% CrI, −0.10 to −0.04) and low-molecular weight phthalate mixtures (β = −0.13; 95% CrI, −0.18 to −0.08) were associated with a decreased MetS score. Most EDC mixtures were associated with elevated proinflammatory proteins, amino acids, and altered glycerophospholipids, which in turn were associated with increased MetS score.

**Conclusions and Relevance:**

This cohort study suggests that prenatal exposure to EDC mixtures may be associated with adverse metabolic health in children. Given the pervasive nature of EDCs and the increase in MetS, these findings hold substantial public health implications.

## Key Points

**Question:**

Is prenatal exposure to mixtures of endocrine-disrupting chemicals (EDC) associated with metabolic dysfunction in children?

**Findings:**

In this cohort study of 1134 mother-child pairs from 6 European countries, prenatal exposures to EDC mixtures, including metals, organochlorine pesticides, polybrominated diphenyl ethers, and perfluoroalkyl substances, were associated with increased metabolic syndrome risk score and altered proinflammatory proteins, amino acids, and glycerophospholipid levels in childhood.

**Meaning:**

These results suggest that exposure to widespread EDC mixtures in pregnancy may be associated with adverse metabolic health in children and contribute to the ongoing surge of metabolic syndrome across the life course.

## Teaser

This cohort study analyzes prenatal exposures to various mixtures of endocrine-disrupting chemicals and their associations with metabolic syndrome and levels of proinflammatory proteins, amino acids, and glycerophospholipids among children in Europe.

## Introduction

Metabolic Syndrome (MetS) represents a cluster of multiple factors associated with increased risk for cardiovascular diseases and type 2 diabetes (T2D), including abdominal obesity, hypertension, insulin resistance and dyslipidemia, affecting 1 in 4 adults worldwide.^1^ Pediatric MetS prevalence ranges from 2% to 10%,^2^ with individual risk factors on the rise.^3,4,5^ Childhood MetS has shown great utility in predicting adult MetS, T2D, and cardiovascular disease,^6,7,8^ even better than individual MetS components.^9^

Exposure to endocrine-disrupting chemicals (EDCs) during fetal development, a critical period of increased susceptibility and programming, may increase the risk of MetS later in life.^10^ EDCs are a class of environmental pollutants with the ability to cross the blood-placenta barrier and interfere with human metabolism and hormonal balance.^10^ These include pesticides, metals, plasticizers such as phthalates and phenols, and other widely used chemicals, including perfluoroalkyl substances (PFAS).^10^

While previous research has examined associations of prenatal EDC exposure with separate components of MetS in children,^11,12^ comprehensive studies on overall cardiometabolic risk remain limited.^13,14,15,16,17,18^ Additionally, only a few studies have assessed the mixture effects of selected classes of EDCs,^16,17,18^ despite widespread exposure to chemical mixtures. Finally, although some associations of prenatal EDC exposure with protein and metabolic signatures in childhood have been described,^19,20^ the internal phenotypes associated with EDC mixtures and underlying MetS pathogenesis are poorly understood.

We utilized a multicenter cohort of 1134 mothers and their children aged 6 to 11 years to conduct the most comprehensive study to date of the association of prenatal EDC mixtures from 9 chemical classes with child MetS risk score. Furthermore, we aimed to identify associated protein and metabolic signatures to unravel underlying mechanisms and altered metabolic pathways.

## Methods

### Study Population

This cohort study used data from the Human Early Life Exposome (HELIX) project and followed the Strengthening the Reporting of Observational Studies in Epidemiology (STROBE) reporting guideline. The HELIX Project is a collaboration between 6 ongoing European longitudinal population-based cohort studies^21^: Born in Bradford (BiB [UK]),^22^ Étude des Déterminants Pré et Postnatals du Développement et de la Santé de l’Enfant (EDEN [France]),^23^ Infancia y Medio Ambiente (INMA [Spain]),^24^ Kaunas Cohort (KANC [Lithuania]),^25^ Norwegian Mother, Father, and Child Cohort Study (MoBa [Norway]),^26^ and Mother and Child Cohort in Crete (RHEA [Greece]).^27^ Approval for the HELIX project was obtained from local ethics committees in each country, and all participating families provided written informed consent. Pregnant women across cohorts were recruited between April 1, 2003, and January 30, 2009. From December 1, 2013, until February 26, 2016, a subcohort of 1301 mother-child pairs was followed-up when the children were aged 6 to 11 years using standardized protocols for clinical examination, interview, and biological sample collection.^28^ Details about the protocol and subcohort inclusion criteria are described elsewhere.^28^ This study included mother-child pairs with measured prenatal EDC exposures and complete data on childhood MetS risk factors, proteins, and metabolites (eFigure 1 in Supplement 1).

### EDCs Exposure Assessment

EDC levels were measured in maternal serum, plasma, whole blood, and urine samples collected during pregnancy or cord blood at birth (eTable 1 in Supplement 1).^29^ A total of 45 compounds from 9 chemical classes were analyzed: 9 metals; 3 organochlorine (OC) pesticides; 5 polychlorinated biphenyls (PCBs); 2 polybrominated diphenyl ethers (PBDEs); 5 PFASs; 7 high-molecular-weight phthalate metabolites (HMWPs), including 4 diethylhexyl phthalate (DEHP) metabolites, 2 di-isononylphthalate (DiNP) metabolites, and 1 metabolite of butyl benzyl phthalate; 3 low-molecular-weight phthalate metabolites (LMWPs); 4 parabens; 3 phenols; and 4 organophosphate (OP) pesticide metabolites. Persistent organic pollutants (OC pesticides, PCBs, PBDEs, and PFAS) and metals were determined in maternal blood except for total mercury which was measured in cord blood in the INMA cohort. Lipophilic compounds (OC pesticides, PCBs, and PBDEs) were corrected for plasma or serum lipid content and expressed in nanograms per gram of lipids. Nonpersistent EDCs (phthalates, parabens, phenols, and OP pesticides) were measured in a spot maternal urine sample, corrected for urine creatinine levels to account for urine dilution and expressed in micrograms per gram of creatinine. Details on laboratories and analytical methods are in eTable 2 in Supplement 1. Quality control, interlaboratory comparison, and limit of detection for each laboratory are available elsewhere.^29^ Values below the limit of detection (0%-30%) were singly imputed using a quantile regression approach for the imputation of left-censored missing data with the R statistical software version 4.3.2 rexposome package (R Project for Statistical Computing).^30^ This fill-in estimation method offers the advantage of inserting values between 0 and the limit of detection while preserving the shape of a normal distribution.

### MetS Risk Score

We calculated a continuous MetS risk score using the score previously validated for children by the European Multicenter Identification and Prevention of Dietary and Lifestyle-Induced Health Effects in Children and Infants study.^31^ Further details are in the eMethods in Supplement 1. We applied the following formula to build the MetS risk score:

*z*-waist circumference + (–*z* high-density lipoprotein cholesterol + *z*-triglycerides)/2 + *z*-insulin + (*z*-systolic blood pressure + *z*-diastolic blood pressure)/2,

where *z *refers to the standardized risiduals. A higher score indicates a higher risk of developing MetS.

### Child Metabolites and Proteins

We used targeted methods to assess metabolite and protein levels in child urine and blood samples collected at the same follow-up visit. Metabolites were assessed at the Imperial College of London (London, UK). A total of 44 urinary metabolites were characterized with ^1^H nuclear magnetic resonance spectroscopy. Serum metabolites were quantified with the liquid chromatography–mass spectrometry metabolomic assay AbsoluteIDQ p180 kit (Biocrates), allowing for the analysis of 177 metabolites, including amino acids, biogenic amines, acylcarnitines, glycerophospholipids, sphingolipids, and sum of hexoses. A total of 35 plasma proteins were determined with 3 Luminex multiplex assays: Cytokines 30-plex, Apoliprotein 5-plex, and Adipokine 15-plex (University Pompeu Fabra Centre for Genomic Regulation Proteomics Unit, Barcelona, Spain). Details about the assessment of children’s metabolites and proteins are available in eAppendix 1 in Supplement 1 and elsewhere.^20^

### Statistical Analysis

Maternal EDCs and child protein and metabolites were log2-transformed to correct skewed distributions. Missing data for all exposures and covariates (0%-54%; eTable 3 in Supplement 1) were imputed using multiple imputations by chained equations, generating 20 imputed data sets, which were combined using Rubin rules in all the subsequent analyses.^32^ Further imputation details are available elsewhere.^33^

Adjusted generalized additive models confirmed no departures from linearity between individual EDCs and MetS risk score (eFigure 2 and eFigure 3 in Supplement 1). We used bayesian weighted quantile sum (BWQS) regressions to assess associations of mixtures of EDCs belonging to each chemical class with MetS score. BWQS estimates a single weighted index summarizing overall exposure to the mixture considering the relative contribution of each exposure within the group mixture using weights.^34^ BWQS characteristics are available in eAppendix 2 in Supplement 1. BWQS regressions were stratified by sex due to potential sex-specific effects of some EDCs.^35,36,37^ We note that sex interactions were not tested due to the absence of interaction testing functions in the BWQS package. Sensitivity analyses to ensure results robustness included (1) single-exposure analyses using linear regressions, correcting for multiple testing with false discovery rate (FDR) and assessment of between-cohort heterogeneity using the *I^2^* statistic of association^38^; (2) mixture analyses for lipophilic compounds, stratifying mothers by gestational weight gain according to the Institute of Medicine guidelines^39^; (3) phthalate mixture analysis, incorporating molar sums of DEHP and DiNP metabolites; (4) metal and persistent mixture analysis, including sum of PCBs, and nonpersistent mixture analysis, incorporating molar sums of DEHP, DiNP, and parabens; and (5) testing main significant mixture associations with a binarized MetS risk outcome, using the 80th percentile as the cutoff.

To identify proteins and metabolites associated with both chemical mixtures and MetS, we first fitted generalized linear regression models between each molecular feature and the MetS risk score, correcting for multiple testing using FDR. Subsequently, we performed BWQS regressions between EDC classes and each associated molecular feature with an FDR *P* value < .05. BWQS model corrections for multiple testing were computed using *P* values derived from bayesian probability of direction, following Makowski et al.^40^ All analyses were conducted in R version 4.3.2.

All statistical models were adjusted for the confounders selected based on previous knowledge and a directed acyclic graph (eFigure 4 in Supplement 1), including subcohort, parental country of birth (both parents native, none or 1 parent native), maternal age, self-reported prepregnancy body mass index (BMI; calculated as weight in kilograms divided by height in meters squared), maternal educational level, parity, maternal smoking in pregnancy, and fish intake in pregnancy. Statistical analysis occurred from October 2022 to July 2023.

## Results

Our study comprised 1134 mother–child pairs (mean [SD] maternal age, 30.7 [4.9] years; 517 female children [45.6%] and 617 male children [54.4%]) (Table). Of all mothers, 574 (50.6%) were highly educated and 506 (44.6%) were nulliparous. The mean (SD) age of children was 7.8 (1.5) years at outcome assessment. The mean (SD) MetS score was −0.1 (2.3), with 341 children (30.1%) classified as high-risk. Maternal prepregnancy BMI was associated with higher child MetS score, and there was some variation in MetS score by cohort (eTable 4 in Supplement 1). Prenatal EDC concentrations are detailed in eTable 5 in Supplement 1. Pearson correlations indicated positive moderate to high correlation within each EDC class, with a few negative correlations within the metals class (eFigure 5 in Supplement 1).

**Table.** Characteristics of the Human Early Life Exposome Subcohort Study Population

Characteristic	Participants, No. (%) (N = 1134)
Parental characteristics	
Subcohort	
BiB (UK)	193 (17.0)
EDEN (France)	144 (12.7)
INMA (Spain)	206 (18.2)
KANC (Lithuania)	196 (17.3)
MoBa (Norway)	205 (18.1)
RHEA (Greece)	190 (16.8)
Family native from the country of the cohort	
At least 1 native parent	1010 (89.1)
No native parent	124 (10.9)
Maternal age at birth, mean (SD), y	30.7 (4.9)
Maternal prepregnancy body mass index, mean (SD)^a^	25.1 (5.0)
Gestational weight gain status (Institute of Medicine criteria)	
Low or adequate	503 (44.4)
Excessive	631 (55.6)
Maternal educational level	
Low	167 (14.7)
Medium	393 (34.7)
High	574 (50.6)
Parity	
Nulliparous	506 (44.6)
Primiparous	421 (37.1)
Multiparous	207 (18.3)
Maternal smoking in pregnancy	
No	966 (85.2)
Yes	168 (14.8)
Maternal fish intake in pregnancy	
<2 times/wk	468 (41.3)
2-4 times/wk	342 (30.2)
>4 times/wk	324 (28.6)
Child characteristics	
Sex	
Female	517 (45.6)
Male	617 (54.4)
Age at assessment, mean (SD), y	7.8 (1.5)
Waist circumference, mean (SD), cm	58.5 (7.6)
Systolic blood pressure, mean (SD), mm Hg	99.1 (11.1)
Diastolic blood pressure, mean (SD), mm Hg	58.3 (9.6)
High-density lipoprotein cholesterol, mean (SD), mg/dL	59.4 (12.4)
Triglycerides, median (IQR), mg/dL	75.3 (59.3-101.0)
Insulin, median (IQR), μIU/mL	317.6 (217.7-552.5)
Metabolic syndrome score, mean (SD)	−0.1 (2.3)
Metabolic syndrome risk group (80th percentile: 1.7 cutoff score)	
Low-risk	793 (69.9)
High-risk	341 (30.1)

Abbreviations: BiB, Born in Bradford; EDEN, Étude des Déterminants Pré et Postnatals du Développement et de la Santé de l’Enfant; INMA, Infancia y Medio Ambiente; KANC, Kaunas cohort; MoBa, Norwegian Mother, Father, and Child Cohort Study; RHEA, RHEA Mother Child Cohort.

SI conversion factors: To convert high-density lipoprotein cholesterol to millimoles per liter, multiply by 0.0259; insulin to picomoles per liter, multiply by 6.945; triglycerides to millimoles per liter, multiply by 0.0113.

^a^ Body mass index was calculated as weight in kilograms divided by height in meters squared.

**Figure 1.**
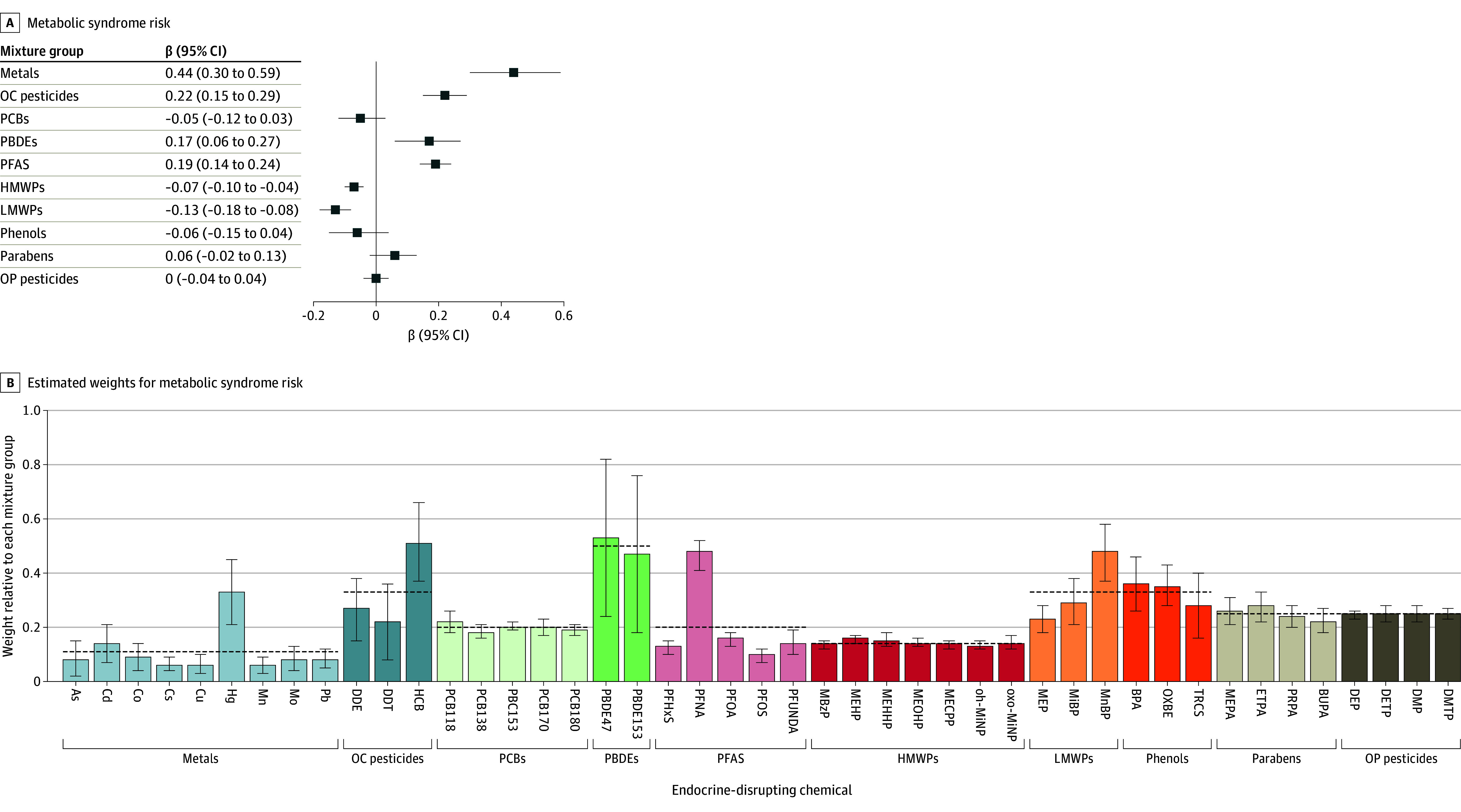
Metabolic Syndrome (MetS) Risk and Estimated Posterior Weights of Exposure Mixture Groups on MetS Risk Score Using the Bayesian Weighted Quantile Sum Regression Panel A shows β coefficient and 95% credible intervals for child MetS per quartile increase in prenatal chemical mixtures. Panel B shows the estimated posterior weights with 95% credible intervals (CrIs; presented as error bars) for MetS risk. Weights represent the relative contribution of each chemical to the overall group association. Within a chemical mixture group, the estimated weights total 1. Dotted horizontal lines indicate expected weights if all chemicals within a group contributed equally to the mixture. All models were adjusted for subcohort, parental country of birth, maternal age, maternal education level, maternal prepregnancy body mass index, parity, maternal smoking in pregnancy, and maternal fish intake in pregnancy. As indicates, inorganic arsenic; BPA, bisphenol A; BUPA, N-butyl paraben; Cd, cadmium; Co, cobalt; Cs, caesium; Cu, copper; DDE, dichlorodiphenyldichloroethylene; DDT, dichlorodiphenyltrichloroethane; DEP, diethyl phthalate; DETP, diethylthiophosphate; DMP, dimethyl phthalate; DMTP, dimethylthiophosphate; ETPA, ethyl paraben; HCB, hexachlorobenzene; Hg, total mercury; HMWPs, high-molecular weight phthalates; LMWPs, low-molecular weight phthalates; MBzP, monobenzylphthalate; MECPP, Mono-(2-ethyl-5-carboxypentyl) phthalate cyclodiphosphate; MEHHP, mono(2-ethyl-5-hydroxyhexyl) phthalate; MEHP, mono-2-ethylhexyl phthalate; MEOHP, mono(2-ethyl-5-oxohexyl) phthalate; MEP, monoethyl phthalate; MEPA, methyl paraben; MiBP, mono-iso-butyl phthalate; Mn, manganese; MnBP, mono-n-butyl phthalate; Mo, molybdenum; OC, organochlorine; oh-MiNP, mono-hydroxy-isononyl phthalate; OP, organophosphate; OXBE, oxybenzone; oxo-MiNP, mono-oxo-isononyl phthalate; Pb, lead; PBDEs, polybrominated diphenyl ethers; PCBs, polychlorinated biphenyls; PFAS, perfluoroalkyl substances; PFHxS, perfluorohexane sulfonate; PFNA, perfluorononanoic acid; PFOA, perfluoro-octanoic acid; PFOS, perfluoro-octane sulfonate; PFUNDA, perfluoroundecanoic acid; PRPA, propyl paraben; and TRCS, triclosan.

Mixture analyses showed increased MetS risk score per 1-quartile increase in prenatal EDC mixture for metals (β = 0.44; 95% credible interval [CrI], 0.30 to 0.59), OC pesticides (β = 0.22; 95% CrI, 0.15 to 0.29), PBDEs (β = 0.17; 95% CrI, 0.06 to 0.27), and PFAS (β = 0.19; 95% CrI, 0.14 to 0.24); while HMWPs and LMWPs were associated with decreased MetS risk score (β for HMWPs = −0.07; 95% CrI, −0.10 to −0.04; β for LMWPs = −0.13; 95% CrI, −0.18 to −0.08) (Figure 1A and eTable 6 in Supplement 1). No association was observed for mixtures of PCBs, phenols, parabens, and OP pesticide metabolites (Figure 1A and eTable 6 in Supplement 1). The main contributor to the metal mixture association was mercury (weight, 0.33). Hexachlorobenzene (HCB; weight, 0.51) and perfluorononanoic acid (PFNA; weight, 0.48) were the primary contributors in the OC pesticides and PFAS mixture associations. Within the LMWP mixture, mono-n-butyl phthalate (MnBP) showed the highest contribution (weight: 0.48), while chemicals within PBDEs and HMWPs had similar weights within each group (Figure 1B and eTable 7 in Supplement 1).

**Figure 2.**
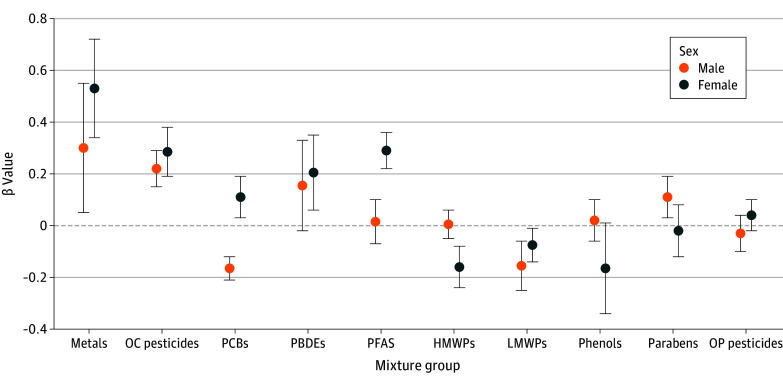
Associations of Prenatal Chemical Mixtures With Metabolic Syndrome (MetS) Score Stratified by Sex The dots denote the β estimate for MetS score per quartile increase in prenatal endocrine-disrupting chemical mixture exposure and the bars denote the 95% credible intervals from bayesian weighted quantile sum regression models. The horizontal dashed line at 0 line indicates the null. All models were adjusted for subcohort, parental country of birth, maternal age, maternal education level, maternal prepregnancy body mass index, parity, maternal smoking in pregnancy, and maternal fish intake in pregnancy. HMWPs indicate high-molecular-weight phthalates; LMWPs, low-molecular-weight phthalates; PBDEs, polybrominated diphenyl ethers; PCBs, polychlorinated biphenyls; PFASs, perfluoroalkyl substances; OC, organochlorine; OP, organophosphate.

When stratified by sex, nonoverlapping 95% CrIs were observed for PCB, PFAS, and HMWP mixtures between both groups (Figure 2). The PCB mixture was associated with higher MetS score in female children (β = 0.11; 95% CrI, 0.03 to 0.19) and lower MetS score in male children (β = −0.17; 95% CrI, −0.21 to −0.12) (Figure 2 and eTable 6 in Supplement 1). Associations of PFAS and HWMPs with MetS score were observed only in female children.

**Figure 3.**
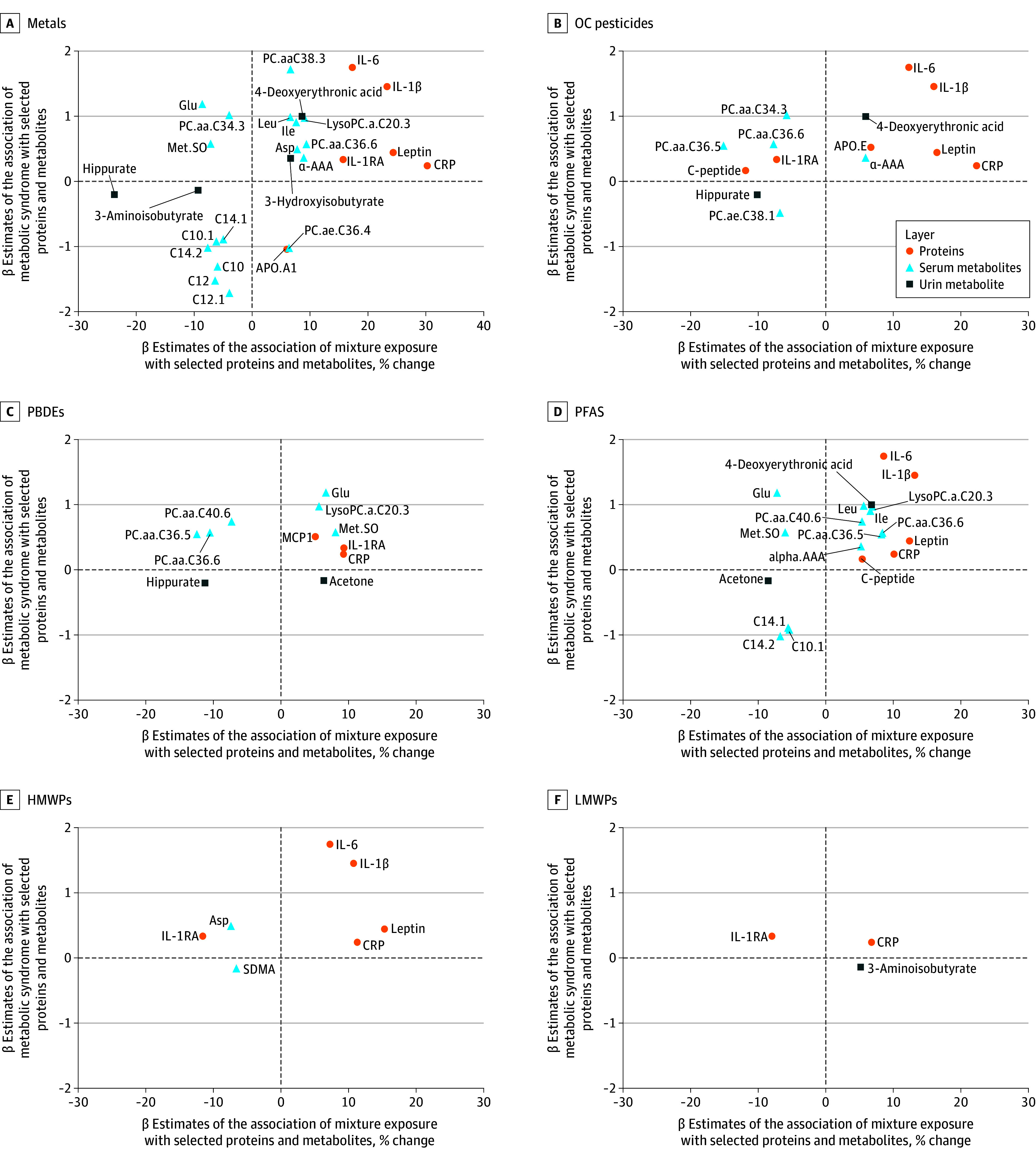
Scatterplot of Selected Proteins and Metabolites Associated With at Least 1 Prenatal Chemical Mixture and Child Metabolic Syndrome (MetS) Risk Score Each point corresponds to a protein or serum or urine metabolites. The x-axis shows the β coefficient of the associations of prenatal mixture with child omics expressed as percent change of omics levels per quartile increase of the exposure mixture (only associations with a % change >5% are shown). The y-axis shows the β coefficient of the associations of child omics with child MetS risk score expressed per doubling of omics levels. This analysis has been restricted to chemical mixtures significantly associated with MetS risk. Dotted vertical and horizontal lines denote the null. All models were adjusted for subcohort, parental country of birth, maternal age, maternal education level, maternal prepregnancy body mass index, parity, maternal smoking in pregnancy, and maternal fish intake in pregnancy. α-AAA indicates alpha-aminoadipic acid; APO, apolipoprotein; Asp, aspartate; C, acylcarnitines; CRP, C reactive protein; HMWPs, high-molecular-weight phthalates; Glu, glutamate; IL, interleukin; Ile, isoleucine; IL-1RA, interleukin 1 receptor antagonist; Leu, leucine; LMWPs, low-molecular-weight phthalates; LysoPC, lysophosphatidylcholines; MCP1, monocyte chemoattractant protein-1; Met.SO, methionine sulfoxide; OCs, organochlorines; PBDEs, polybrominated diphenyl ethers; PC, phosphatidylcholine; PFASs, perfluoroalkyl substances; SDMA, symmetric dimethylarginine.

A total of 14 plasma proteins, 110 serum metabolites and 10 urine metabolites were cross-sectionally associated with child MetS risk score (eTables 8-10 in Supplement 1). Figure 3 depicts associations of these metabolites and proteins with the prenatal chemical mixtures (for visualization purpose, only those with a percent change >5% are shown) and child MetS risk score. The number of molecular markers related to both MetS score and the mixture ranged from 43 for the LMWP mixture to 109 for the PFAS mixture (eTables 11-13 in Supplement 1). All prenatal mixtures were associated with elevated serum levels of C-reactive protein (CRP) and at least 2 of the following proteins, which were all associated with higher MetS score: interleukin (IL)-1β, IL-6, IL-1RA, and leptin (Figure 3, eTable 8, and eTable 11 in Supplement 1). Metals and persistent chemicals were associated with increased serum levels of α-aminoadipic acid (α-AAA), leucine, isoleucine, and valine, which also were associated with an increased MetS score. The same set of mixtures was associated with altered diacyl chain phosphatidylcholine levels, primarily associated with higher MetS score. Metals and PFAS were associated with decreased acylcarnitines, which were associated with lower MetS score (Figure 3, eTable 9, and eTable 12 in Supplement 1). Metals and persistent chemicals (except for PBDEs) were associated with higher urine concentrations of 4-deoxyerythronic acid and 3-hydroxisobutyrate, which were also associated with higher MetS score. Conversely, all mixtures, except for LMWPs, were associated with lower child urine hippurate, which was associated with lower MetS score (Figure 3, eTable 10, and eTable 13 in Supplement 1).

### Sensitivity Analyses 

Single-exposure analyses showed an association of prenatal MnBP levels with decreased child MetS risk score (β = −0.17; 95% CrI, −0.34 to −0.01), but no other associations were observed (eTable 14 in Supplement 1). Between-cohort heterogeneity was not observed (*I^2^* values close to 0%). The association of OC pesticide mixtures with child MetS risk was notable only in children whose mothers had low or adequate weight gain during pregnancy (β = 0.32; 95% CrI, 0.23 to 0.40), whereas no clear differences were observed by weight gain categories for other classes of lipophilic chemicals (eTable 15 in Supplement 1). Associations of whole phthalate mixture, nonpersistent chemical mixture, and child MetS risk were comparable to those found with the LMWPs mixture (eTable 16 in Supplement 1). Notably, the metals and persistent chemical mixture exhibited larger associations than separate chemical groups (β = 0.63; 95% CrI, 0.47 to 0.78) (eTable 17 in Supplement 1). Associations remained consistent when using the dichotomous MetS risk outcome (eTable 18 in Supplement 1).

## Discussion

In this multicenter cohort study of European mothers and their children, maternal exposure to mixtures of metals, OC pesticides, PBDEs, and PFAS during pregnancy was associated with an increased MetS risk score in childhood, while phthalate mixtures were associated with a lower MetS score. Our results suggest sex-specific associations for certain chemicals and identify molecular signatures in childhood associated with both prenatal EDC exposure and child MetS risk. Notably, associations of metals and persistent chemicals with MetS closely resemble those previously observed with nonalcoholic fatty liver disease risk,^41^ which contributes to increasing evidence supporting the relationship between both disorders.

To our knowledge, this study represents the first comprehensive evaluation of associations of prenatal exposure to mixtures of a wide range of EDC classes with MetS risk, and protein and metabolite profiles in childhood. Our use of state of the art mixture methods revealed associations not evident in single exposure models, highlighting the importance of evaluating health risks associated with EDC mixtures. The use of an aggregate MetS score offered a more comprehensive approach compared with isolated risk factors, capturing the overall metabolic effect better. By identifying child molecular phenotypes associated with EDC mixtures and underlying MetS, this study may support future early identification of EDC-exposed pediatric populations at risk for MetS development. Furthermore, the sample size of more than 1000 mother-child pairs enabled stratification and comparison of associations between male and female children. Previously, only 1 study^18^ assessed the association of a prenatal metal mixture with child MetS risk, observing a null association. However, this mixture did not include mercury,^18^ a high priority pollutant that has been suggested to elicit oxidative stress and inflammation.^42,43^ We found that mercury was the metal with the highest contribution to increasing child MetS. Our results corroborate the adverse metabolic health associated with prenatal mercury exposure previously found in the HELIX project^15^ and other epidemiological studies.^43^

The obesogenic effects of in utero exposure to persistent chemicals, including OC pesticides, PBDEs, and PFAS have been extensively reported.^11^ However, only a few studies^13,14,16^ have examined their association with MetS risk in childhood. We observed HCB to be the main contributor of the OC pesticides mixture association and PFNA to be the main contributors of the PFASs mixture association. These findings align with earlier studies using the Spanish INMA cohort,^13,14^ which found that prenatal HCB and PFNA was associated with higher MetS risk in adolescence and childhood. Of interest, PFNA was also the main contributor to prenatal PFAS mixture associations related to higher liver enzymes and liver injury in children in HELIX.^41,44^

Phthalates, as nonpersistent EDCs quickly metabolized and excreted in urine, have been shown in experimental studies^45,46^ to disrupt fetal programming of cardiovascular function and adipogenesis, predisposing to offspring MetS pathogenesis.^12^ Surprisingly, we found that prenatal exposure to phthalates was associated with a decreased child MetS risk score. Similarly, a study^17^ with over 2000 Chinese mother-child pairs reported inconsistent associations of phthalate mixture exposure with MetS risk in childhood, depending on the exposure timing in pregnancy. We found no associations with MetS score for the phenols, parabens, and OP pesticide metabolite mixtures, even though single exposure studies have documented associations with components of MetS, especially for bisphenol A.^10,11,47^ The reason for such inconsistencies may be potential measurement error given the exposure assessment in a single urine sample.^48^

Our results suggest sex-specific metabolic disruption, with females being more susceptible to PFAS and PCBs exposure. This could be due to their interference with sex steroid hormone pathways,^49,50,51^ which in the case of PFAS, has been observed in human fetuses.^52^

Our analysis of molecular markers unveiled proteins and metabolites associated with both prenatal EDC exposure and MetS development. Most mixtures were associated with upregulated cytokines, CRP, and leptin, all of them being proinflammatory proteins produced and released by the adipose tissue relevant to MetS pathogenesis.^53,54^ CRP has also shown to be associated with increased risk of MetS, T2D, and cardiovascular disease in healthy adults.^55^ Our findings, using an exposure-mixture approach in a sensitive time-window as pregnancy, corroborate prior in vitro*, *in vivo, and epidemiological studies^56,57,58,59,60,61,62^ that showed similar associations with individual metals, persistent chemicals, and phthalate exposure, including earlier HELIX studies^15,63^ assessing prenatal mercury and PFAS exposure.

At the metabolite level, we observed elevated levels of serum branched chained amino acids, α-AAA, urinary 4-deoxyerythronic acid, 3-hydroxisobutyrate, and dysregulation in diacyl chain phosphatidylcholines associated with prenatal metals and persistent chemicals mixtures and increased MetS score. Branched chained amino acids, well-known regulators of glucose and lipid metabolism,^64^ and α-AAA have been shown to be associated with MetS risk components in healthy adults before disease onset.^65,66,67,68^ Consistently, 4-deoxyerythronic levels were associated with a higher BMI in childhood in the HELIX study.^69^ 3-Hydroxyisobutyrate, whose fermentation by gut bacteria may lead to the production of short-chain fatty acids regulating overall metabolic activity,^70^ has also been associated with to obesity, insulin resistance, and T2D.^71^ Perturbations in lipid metabolites associated with OC pesticides and PFAS exposure were noted in previous studies with children,^72,73^ and with prenatal exposure in children.^44^ Diacyl chain phosphatidylcholines are crucial for the liver’s release of triglyceride-rich, very low density lipoprotein particles and high-density lipoprotein,^74^ consistent with our findings and with metabolomics studies in patients with T2D^75^ and obesity.^76^ Acylcarnitines transport fatty acids to mitochondria for oxidation,^77^ and elevated levels may be associated with T2D and MetS risk.^78,79^ Odd-chain acylcarnitines, whose main source is circulating fatty acids from diet,^80^ were associated with decreased MetS risk score in childhood. We speculate that these results may be partly due to differences in diet and fasting status.^80^ Chemical mixtures were associated with lower urine hippurate levels. Hippurate, originating from dietary polyphenols metabolism,^81^ has been associated with increased gut microbiome diversity and reduced MetS risk,^82^ aligning with our findings.

### Limitations

Our study has several limitations. First, potential measurement error in assessing highly variable nonpersistent chemicals^83^ with attenuation bias reached as high as 80%.^84^ Second, lipids and insulin were analyzed from blood taken after only 3 hours of fasting, which may have reduced the precision of the MetS risk score. Third, although we used a longitudinal study design, proteins and metabolomic biomarkers in children were assessed at the same time point as the MetS score, limiting the ability to disentangle any mediating effect of the observed features of the EDC-MetS associations. Fourth, no multiordered chemical-chemical interactions were considered in this study, hindering the possibility of detecting potential toxicological interactions among EDCs.^85^ Fifth, because our focus was on EDCs exposure during fetal programming, childhood exposures were not considered, rendering it challenging to discern effects across exposure windows. For some of our EDC classes, further caution is warranted because exposure data were missing (ie, the chemicals were not analyzed) for a relatively large proportion of subjects; for example, PBDEs were missing in 51% to 54% of participants. To address this, we used multiple imputations to minimize bias that would result from deleting participants with missing data, thereby avoiding loss of power.^86,87^ We opted not to conduct complete-case analyses due to the limited sample size (less than 400 participants), which would not allow for a meaningful comparison with our imputed data sets. Future studies incorporating repeated urine samples during pregnancy,^88^ and novel statistical tools combining mixtures with mediation and interaction analysis are needed for a deeper understanding of EDC metabolic effects.

## Conclusions

This large, population-based cohort study suggests that prenatal exposure to EDCs mixtures, particularly metals, OC pesticides, PBDEs, and PFASs, may be associated with adverse metabolic health in childhood. These findings advance our limited understanding of metabolic effects of EDC mixtures in early life and can inform more efficient early-life prevention and intervention strategies to address rising trends in MetS across the life course.

## Notes

Supplement 1. **eFigure 1.** Flow Chart Describing Population Included in the Study **eTable 1.** Biological Matrices and Time of Collection During Pregnancy for EDC Assessment **eTable 2.** Analytical Methods and Laboratories for Prenatal EDCs, Lipids and Creatinine Assessment **eMethods.** MetS Risk Score **eAppendix 1.** Measurement of Metabolites and Protein Levels in Childhood **eTable 3.** Percentage of Missings in the Chemicals’ Exposures and Covariates **eFigure 2.** Generalized Additive Models of Prenatal Exposure to Metals and Persistent Chemicals and Child Metabolic Syndrome Risk Score to Test Linearity Prior Using BWQS **eFigure 3.** Generalized Additive Models of Prenatal Exposure to Nonpersistent Chemicals and Child Metabolic Syndrome Risk Score to Test Linearity Prior Using BWQS **eAppendix 2.** Bayesian Weighted Quantile Sum (BWQS) Regression Characteristics **eFigure 4.** Directed Acyclic Graph (DAG) Elucidating Exposure-Outcome Association **eTable 4.** Cofounder-Only Model for MetS Score **eTable 5.** Summary Statistics of Log2-Transformed Prenatal EDC Concentrations **eFigure 5.** Correlation Plot Between EDC Exposures **eTable 6.** BWQS Models for MetS for All the Study Population and Stratified by Sex **eTable 7.** Estimated Posterior Weights of Exposure Mixture Groups on MetS Using BWQS Models for All the Study Population and Stratified by Sex **eTable 8.** Adjusted Associations Between Childhood Proteins and Child MetS Risk Score **eTable 9.** Adjusted Associations Between Childhood Serum Metabolites and Child MetS Risk Score **eTable 10.** Adjusted Associations Between Childhood Urine Metabolites and Child MetS Risk Score **eTable 11.** Adjusted Associations Between Prenatal EDC Mixtures and Child Proteins Using BWQS **eTable 12.** Adjusted Associations Between Prenatal EDC Mixtures and Child Serum Metabolites Using BWQS **eTable 13.** Adjusted Associations Between Prenatal EDC Mixtures and Child Urine Metabolites Using BWQS **eTable 14.** Environmental-Wide Association Study (ExWAS) of Prenatal Individual EDCs and Child MetS **eTable 15.** Sensitivity Analysis. BWQS Models of Lipophilic Mixture Groups and MetS Stratified by Gestational Weight Gain Category **eTable 16**. Sensitivity Analyses. BWQS Models of Phthalate and Non-Persistent Chemical Mixtures **eTable 17.** Sensitivity Analyses. BWQS Models of Metals and Persistent Chemicals Mixture **eTable 18.** Sensitivity Analyses. BWQS Models for Dichotomous MetS Risk (Low vs High) for All the Study Population
eReferences

Supplement 2.
Data Sharing Statement
