# Supplementary material for: Prenatal Exposure to Chemical Mixtures and Metabolic Syndrome Risk in Children
Source: JAMA Netw Open. 2024 May 23;7(5):e2412040. doi: 10.1001/jamanetworkopen.2024.12040 (PMC11117089; doi:10.1001/jamanetworkopen.2024.12040)
Supplement: Supplement 1. — eFigure 1. Flow Chart Describing Population Included in the Study eTable 1. Biological Matrices and Time of Collection During Pregnancy for EDC Assessment eTable 2. Analytical Methods and Laboratories for Prenatal EDCs, Lipids and Creatinine Assessment eMethods. MetS Risk Score eAppendix 1. Measurement of Metabolites and Protein Levels in Childhood eTable 3. Percentage of Missings in the Chemicals’ Exposures and Covariates eFigure 2. Generalized Additive Models of Prenatal Exposure to Metals and Persistent Chemicals and Child Metabolic Syndrome Risk Score to Test Linearity Prior Using BWQS eFigure 3. Generalized Additive Models of Prenatal Exposure to Nonpersistent Chemicals and Child Metabolic Syndrome Risk Score to Test Linearity Prior Using BWQS eAppendix 2. Bayesian Weighted Quantile Sum (BWQS) Regression Characteristics eFigure 4. Directed Acyclic Graph (DAG) Elucidating Exposure-Outcome Association eTable 4. Cofounder-Only Model for MetS Score eTable 5. Summary Statistics of Log2-Transformed Prenatal EDC Concentrations eFigure 5. Correlation Plot Between EDC Exposures eTable 6. BWQS Models for MetS for All the Study Population and Stratified by Sex eTable 7. Estimated Posterior Weights of Exposure Mixture Groups on MetS Using BWQS Models for All the Study Population and Stratified by Sex eTable 8. Adjusted Associations Between Childhood Proteins and Child MetS Risk Score eTable 9. Adjusted Associations Between Childhood Serum Metabolites and Child MetS Risk Score eTable 10. Adjusted Associations Between Childhood Urine Metabolites and Child MetS Risk Score eTable 11. Adjusted Associations Between Prenatal EDC Mixtures and Child Proteins Using BWQS eTable 12. Adjusted Associations Between Prenatal EDC Mixtures and Child Serum Metabolites Using BWQS eTable 13. Adjusted Associations Between Prenatal EDC Mixtures and Child Urine Metabolites Using BWQS eTable 14. Environmental-Wide Association Study (ExWAS) of Prenatal Individual EDCs and Child MetS eTable 15. Sens [file jamanetwopen-e2412040-s001.pdf]

Supplementary Online Content

Güil-Oumrait N, Stratakis N, Maitre L. Prenatal exposure to chemical mixtures and metabolic syndrome risk in European children. *JAMA Netw. Open*. 2023;7(5):e2411735. doi: 10.1001/jamanetworkopen.2024.11735

eFigure 1. Flow Chart Describing Population Included in the Study
eTable 1. Biological Matrices and Time of Collection During Pregnancy for EDC Assessment
eTable 2. Analytical Methods and Laboratories for Prenatal EDCs, Lipids and Creatinine Assessment
eMethods. MetS Risk Score
eAppendix 1. Measurement of Metabolites and Protein Levels in Childhood
eTable 3. Percentage of Missings in the Chemicals' Exposures and Covariates
eFigure 2. Generalized Additive Models of Prenatal Exposure to Metals and Persistent Chemicals and Child Metabolic Syndrome Risk Score to Test Linearity Prior Using BWQS
eFigure 3. Generalized Additive Models of Prenatal Exposure to Nonpersistent Chemicals and Child Metabolic Syndrome Risk Score to Test Linearity Prior Using BWQS.
eAppendix 2. Bayesian Weighted Quantile Sum (BWQS) Regression Characteristics
eFigure 4. Directed Acyclic Graph (DAG) Elucidating Exposure-Outcome Association
eTable 4. Cofounder-Only Model for MetS Score
eTable 5. Summary Statistics of Log_2-Transformed Prenatal EDC Concentrations
eFigure 5. Correlation Plot Between EDC Exposures
eTable 6. BWQS Models for MetS for All the Study Population and Stratified by Sex
eTable 7. Estimated Posterior Weights of Exposure Mixture Groups on MetS Using BWQS Models for All the Study Population and Stratified by Sex
eTable 8. Adjusted Associations Between Childhood Proteins and child MetS Risk Score
eTable 9. Adjusted Associations Between Childhood Serum Metabolites and Child MetS Risk Score
eTable 10. Adjusted Associations Between Childhood Urine Metabolites and Child MetS Risk Score
eTable 11. Adjusted Associations Between Prenatal EDC Mixtures and Child Proteins Using BWQS
eTable 12. Adjusted Associations Between Prenatal EDC Mixtures and Child Serum Metabolites Using BWQS
eTable 13. Adjusted Associations Between Prenatal EDC Mixtures and Child Urine Metabolites Using BWQS
eTable 14. Environmental-Wide Association Study (ExWAS) of Prenatal Individual EDCs and Child MetS
eTable 15. Sensitivity Analysis. BWQS Models of Lipophilic Mixture Groups and MetS Stratified by Gestational Weight Gain Category
eTable 16. Sensitivity Analyses. BWQS Models of Phthalate and Non-Persistent chemical mixtures
eTable 17. Sensitivity Analyses. BWQS Models of Metals and Persistent Chemicals Mixture
eTable 18. Sensitivity Analyses. BWQS Models for Dichotomous MetS Risk (Low vs High) for All the Study Population

This supplemental material has been provided by the authors to give readers additional information about their work.

© 2024 Güil-Oumrait N et al. *JAMA Network Open.*

eFigure 1. Flow Chart Describing Population Included in the Study

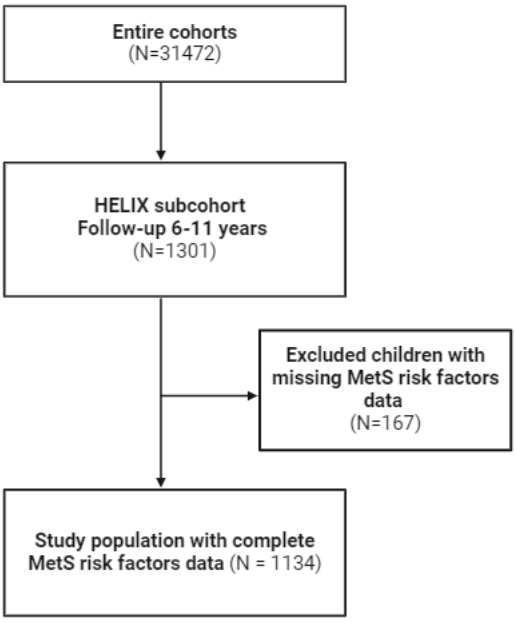

Abbreviations: HELIX, Human Early Life Exposome; MetS, Metabolic Syndrome.

© 2024 Güil-Oumrait N et al. *JAMA Network Open.*

eTable 1. Biological Matrices and Time of Collection During Pregnancy for EDC Assessment

Cohort	Metals	OC pesticides, PCBs and PBDEs	PFASs	Phthalates metabolites	Phenols	OP pesticide metabolites	Gestational week (mean (SD)) at blood collection	Gestational week (mean (SD)) at urine collection
BiB	Whole blood	Serum/ Plasma	Serum/ Plasma	Urine	Urine	Urine	26.6 (1.4)	26.6 (1.4)
EDEN	Whole blood	Serum	Serum	Urine	Urine	Urine	26.1 (1.2)	26.1 (1.2)
INMA	Cord whole blood (Hg)	Serum	Plasma	Urine	Urine	Urine	13.7 (2.0)	34.2 (1.3)
KANC	Whole blood	-	Whole blood	-	-	-	39.4 (1.3)	-
MOBA	Whole blood	Plasma	Plasma	Urine	Urine	Urine	18.7 (0.9)	18.7 (0.9)
RHEA	Whole blood	Serum	Plasma	Urine	Urine	Urine	14.1 (3.7)	14.1 (3.7)

Abbreviations: BiB, Born in Bradford; EDEN, Étude des Déterminants Pré et Postnatals du Développement et de la Santé de l'Enfant; INMA, Infancia y Medio Ambiente; KANC, Kaunas Cohort; MOBA, the Norwegian Mother, Father and Child Cohort Study; RHEA, Mother-Child Cohort in Crete; SD, standard deviation; OC, organochlorine compound; OP, organophosphate; PBDEs, polybrominated diphenyl ethers; PCBs, polychlorinated biphenyls; PFASs, per- and polyfluoroalkyl substances.

© 2024 Güil-Oumrait N et al. *JAMA Network Open.*

eTable 2. Analytical Methods and Laboratories for Prenatal EDCs, Lipids and Creatinine Assessment

Biomarker	Cohort	Laboratory	Analytical method	Reference
Metals	BIB, EDEN, KANC, MOBA, RHEA	ALS Scandinavia (Sweden)	ICP-SFMS	Rodushkin et al. (2000)[8]
	INMA	Hg; Laboratorio de Salud Pública de Alava (Spain)	AAS	Ramon et al. (2001)[9]
OCs and PCBs	BIB, EDEN, MOBA	NIPH (Norway)	GC-MS/MS	Modified in Caspersen et al. (2016)[10]
	INMA	Laboratorio de Salud Pública de Guipúzcoa (Spain)	GC-MS	Goñi et al. (2007)[11]
	RHEA	National Institute for Health and Welfare, Chemical Exposure Unit, Kuopio (Finland)	GC-MS	Koponen et al. (2013)[12]
PBDEs	BIB, EDEN, KANC, INMA, MOBA	NIPH (Norway)	GC-MS/MS	Modified in Caspersen et al. (2016)[10]
	RHEA	National Institute for Health and Welfare, Chemical Exposure Unit, Kuopio (Finland)	GC-MS/MS	Koponen et al. (2013)[12]
PFASs	BIB, EDEN, KANC, RHEA, MOBA	NIPH (Norway)	Online column-switching LC–MS/MS	Haug et al. (2009)[13] for serum and plasma and Poothong et al. (2017)[14] for whole blood
	INMA	Institute for Occupational Medicine, RWTH Aachen University (Germany)	Online column-switching LC–MS/MS	Manzano-Salgado et al. (2015) [15]
Phthalate metabolites	BIB, EDEN, KANC, RHEA, MOBA	NIPH (Norway)	Online column-switching LC–MS/MS	Sabaredzovic et al. (2015)[16]
	INMA	Bioanalysis Research Group at the Hospital del Mar Medical	HPLC-MS	Valvi et al. (2014)[17]

© 2024 Güil-Oumrait N et al. *JAMA Network Open.*

		Research Institute (Barcelona)		
Phenols	BIB, KANC, INMA, RHEA, MOBA	NIPH (Norway)	Online column-switching LC–MS/MS	Sakhi et al. (2018)[18]
	EDEN	National Center for Environmental Health laboratory at the CDC (US)	Online column-switching LC–MS/MS	Philippat et al. (2011)[19]
OP pesticide metabolites	BIB, EDEN, KANC, INMA, RHEA, MOBA	NIPH (Norway)	UHPLC-TOFMS	Cequier et al. (2016)[20]
Lipids	BIB, EDEN, KANC, MOBA	Fürst Medical Analysis Laboratory (Norway)	ADVIA® Chemistry XPT System, and the FS kit from DiaSys was used to measure concentrations of phospholipids.	
	INMA	Laboratorio de Salud Pública de Bizkaia (Spain)	Cobas Mira self-analyzer (Roche Diagnostic, Basel, Switzerland) using an Enzymatic-Colorimetric method with Spinreact reagents	
	RHEA	Medicon Hellas SA, Gerakas (Greece)	Standard enzymatic method	
Creatinine	BIB, KANC, MOBA, RHEA	Fürst Medical Analysis Laboratory (Norway)	AU680 Chemistry System from Beckman Coulter using DRI® Creatinine-Detect® Test	
	INMA	Echevarne Laboratory of Barcelona (Spain)	Jaffé method - Beckman Coulter© AU5400	
	EDEN	National Center for Environmental Health laboratory at the CDC (US)	Enzymatic reaction using a Roche Hitachi 912 chemistry analyzer (Roche Hitachi, Basel, Switzerland)	

Abbreviations: AAS, thermal decomposition, amalgamation and atomic absorption spectrometry; BiB, Born in Bradford; CDC, Centers for Disease Control and Prevention; EDEN, Étude des Déterminants Pré et Postnatals du Développement et de la Santé de l'Enfant; GC-MS/MS, gas chromatography coupled with tandem mass spectrometry; GC-MS, gas chromatography mass spectrometry; HPLC-MS, ultra-performance liquid chromatography coupled to mass spectrometry; ICP-SFMS, conductively coupled plasma-sector field mass spectrometry; INMA, Infancia y Medio Ambiente; KANC, Kaunas Cohort; LC-MS/MS, liquid chromatography-tandem mass spectrometry; MOBA, the Norwegian Mother, Father and Child Cohort Study; NIPH, Norwegian Institute of Public Health; OCs, organochlorine compounds; OPs, organophosphates; PBDEs, polybrominated diphenyl ethers; PCBs, polychlorinated biphenyls; PFAS, perfluoroalkyl substances; RHEA, Mother-Child Cohort in Crete; UHPLC-TOFMS, ultra-performance liquid chromatography coupled to time-of-flight mass spectrometry.

© 2024 Güil-Oumrait N et al. *JAMA Network Open.*

eMethods. MetS Risk Score

All components of MetS risk score were measured during the 6-11-year follow-up visit using a common protocol[1]. Blood samples were collected at the end of the clinical examination after a median fasting time of 3.3 hours (5th – 95th percentile: 2.2 – 5.9 hours). High-density lipoprotein cholesterol (HDL-C) and triglyceride levels were analysed with homogeneous enzymatic colorimetric methods in the MODULAR ANALYTICS system (Roche Diagnostics), and insulin levels were determined using the human adipokine 15-plex magnetic panel (Life Technologies) (eMethods 2). We generated z-scores standardised by age and sex for all MetS components. Blood pressure was additionally standardized for height[2] and triglyceride levels were long-transformed to handle the skewed distribution.

© 2024 Güil-Oumrait N et al. *JAMA Network Open.*

eAppendix 1. Measurement of Metabolites and Protein Levels in Childhood

The description of the measurement of child metabolites and protein levels has been obtained from Maitre et al. 2022[3] and presented here verbatim.

Serum metabolites

"The AbsoluteIDQTM p180 kit was chosen for serum analysis as it is a standardised, targeted LCMS/MS assay, widely used for large-scale epidemiology studies and its inter-laboratory reproducibility has been demonstrated by several independent laboratories [4] . Serum samples were quantified using the AbsoluteIDQTM p180 kit following the manufacturer's protocol (User Manual UM_p180_AB_SCIEX_9, Biocrates Life Sciences AG) using LC-MS/MS; an Agilent HPLC 1100 liquid chromatography coupled to a SCIEX QTRAP 6500 triple quadrupole mass spectrometer. A full description of the HELIX metabolomics methods and data can be found elsewhere[5]. Briefly, the kit allows for the targeted analysis of 188 metabolites in the classes of amino acids, biogenic amines, acylcarnitines, glycerophospholipids, sphingolipids and sum of hexoses, covering a wide range of analytes and metabolic pathways in one targeted assay. The kit consists of a single sample processing procedure, with two separate analytical runs, a combination of liquid chromatography (LC) and flow injection analysis (FIA) coupled to tandem mass spectrometry (MS/MS). Isotopically labelled and chemically homologous internal standards were used for quantification. The AbsoluteIDQ p180 data of serum samples were acquired in 18 batches. Every analytical batch, in a 96-well plate format, included up to 76 randomised cohort samples. Also in every analytical batch, three sets of quality control samples were included, the NIST SRM 1950 plasma reference material (in 4 replicates), a commercial available serum QC material (CQC in 2 replicates, SeraLab, S-123-M-27485) and the QCs provided by the manufacturer in three concentration levels. The NIST SRM 1950 reference was used as the main quality control sample for the LC-MS/MS analysis. Coefficients of variation (CVs) for each metabolite were calculated based on the NIST SRM 1950 and also the limits of detection (LODs) were also used to assess the analytical performance of individual metabolites. Metabolite exclusion was based on a metabolite variable meeting two conditions: (1) CV of over 30% and (2) over 30% of the data are below LOD. Eleven out of the 188 serum metabolites detected were excluded as a result, leaving 177 serum metabolites to be used for further statistical analysis. The mean coefficient of variation across the 177 LC-MS/MS detected serum metabolites was 16%. We also excluded one HELIX sample, which was hemolyzed. Concentration levels were log2 transformed."

Urinary metabolites

"Two urine samples, representing last night-time and first morning voids, were collected on the evening and morning before the clinical examination, kept in a fridge and transported in a temperature-controlled environment, and aliquoted and frozen within 3 h of arrival at the clinics. They were subsequently pooled to generate a more representative sample of the last 24 h for metabolomic analysis. Urinary metabolic profiles were acquired using 1H NMR spectroscopy according to [5]. In brief onedimensional 600 MHz 1H NMR spectra of urine samples from each cohort were acquired on the same Bruker Avance III spectrometer operating at 14.1 Tesla within a period of 1 month. The spectrometer was equipped with a Bruker SampleJet system, and a 5-mm broad-band inverse configuration probe maintained at 300K. Prior to analysis, cohort samples were randomised. Deuterated 3-(trimethylsilyl)-[2,2,3,3-d4]-propionic acid sodium salt (TSP) was used as internal reference. Aliquots of the study pooled quality control (QC) sample were used to monitor analytical performance throughout the run and were analysed at an interval of every 23 samples (i.e. 4 QC samples per well plate). The 1H NMR spectra were acquired using a standard

© 2024 Güil-Oumrait N et al. *JAMA Network Open.*

onedimensional solvent suppression pulse sequence. Forty-four metabolites were identified and quantified as described (Supplementary Data 1H) [5]. The urinary NMR showed excellent analytical performance, the mean coefficient of variation across the 44 NMR detected urinary metabolites was 11%. Data was normalized using the median fold change normalization method30, which takes into account the distribution of relative levels of all 44 metabolites compared to the reference sample in determining the most probable dilution factor. An offset of ½ of the minimal value was applied and then concentration levels were expressed as log2".

Plasma proteins

"Plasma protein levels were assessed using the antibody-based multiplexed platform from Luminex. Three kits targeting 43 unique candidate proteins were selected (Thermo Fisher Scientifics, USA): Cytokines 30-plex (Catalog Number (CN): LHC6003M), Apoliprotein 5-plex (CN: LHP0001M) and Adipokine 15-plex (CN: LHC0017M). All samples were randomized and blocked by cohort prior measurement. For quantification, an 8- point calibration curve per plate was performed with protein standards provided in the Luminex kit and following procedures described by the vendor. Commercial heat inactivated, sterile-filtered plasma from human male AB plasma (Sigma-Aldrich, USA) was used as constant samples to control for intra- and inter-plate variability. Four control samples were added per plate. All samples, including controls, were diluted ½ for the 30-plex kit, ¼ for the 15-plex kit and 1/2500 for the 5-plex kit. Raw intensities obtained with the xMAP and Luminex system for each plasma sample were converted to pg/ml using the calculated standard curves of each plate and accounting for the dilutions made prior measurement. The percentages of coefficients of variation (CV%) for each protein by plate ranged from 3% to 36%. The limit of detection (LOD) and the lower and upper limit of quantification (LOQ1 and LOQ2, respectively) were estimated by plate, and then averaged. Only proteins with >30% of measurements in the linear range of quantification were kept in the database and the others were removed. Seven proteins were measured twice (in two different multiplex kits). We kept the measure with higher quality. The 36 proteins that passed the quality control criteria mentioned above were log2 transformed[5]. Then, the plate batch effect was corrected by subtracting the plate specific average for each protein minus the overall average of all plates for that protein. After that, values below the LOQ1 and above the LOQ2 were imputed using a truncated normal distribution implemented in the truncdist R v1.0-2 package[6]. Twenty samples were excluded due to having ten or more proteins out of the linear range of quantification."

© 2024 Güil-Oumrait N et al. *JAMA Network Open.*

eTable 3. Percentage of Missings in the Chemicals' Exposures and Covariates

**Covariates**	**% of missings**
Subcohort	0%
Family native from the country of the cohort	2.1%
Maternal age at birth	1.1%
Maternal prepregnancy BMI	1.8%
Gestational weight gain	11.9%
Maternal educational level	3.3%
Parity	2.3%
Maternal smoking in pregnancy	2.7%
Maternal fish intake in pregnancy	30.1%
**Chemicals**	**% of missings**
As	35.5%
Cd	35.5%
Co	35.5%
Cs	35.5%
Cu	35.5%
Hg	20.2%
Mn	35.5%
Mo	35.5%
Pb	35.5%
DDE	20.1%
DDT	20.1%
HCB	20.1%
PCB-118	38.7%
PCB-138	21.0%

© 2024 Güil-Oumrait N et al. *JAMA Network Open.*

PCB-153	21.0%
PCB-170	38.7%
PCB-180	21.0%
PBDE-47	50.9%
PBDE-153	54.0%
PFHxS	4.4%
PFNA	4.4%
PFOA	4.4%
PFOS	4.4%
PFUNDA	21.3%
MBzP	17.7%
MEHP	18.0%
MEHHP	17.7%
MEOHP	17.6%
MECPP	32.1%
oh-MiNP	32.0%
oxo-MiNP	32.0%
MEP	18.4%
MiBP	17.7%
MnBP	17.6%
BPA	18.0%
OXBE	17.9%
TRCS	17.9%
MEPA	36.1%
ETPA	35.9%
PRPA	18.1%
BUPA	18.0%
DEP	18.2%
DETP	21.8%

© 2024 Güil-Oumrait N et al. *JAMA Network Open.*

DMP	18.3%
DMTP	18.0%
Abbreviations: As, inorganic arsenic; BPA, bisphenol A; BMI, body mass index; BUPA, N-butyl paraben; Cd, cadmium; Co, cobalt; Cs, caesium; Cu, copper; DDE, dichlorodiphenyldichloroethylene; DDT, dichlorodiphenyltrichloroethane; DEP, diethyl phosphate; DETP, diethylthiophosphate; DMP, dimethyl phosphate; DMTP, dimethylthiophosphate; ETPA, ethyl paraben; HCB, hexachlorobenzene; Hg, mercury; MBzP, monobenzylphthalate; MECPP,mono(2-ethyl-5-carboxypentyl) phthalate; MEHHP, mono(2-ethyl-5-hydroxyhexyl) phthalate; MEHP, mono-2-ethylhexyl phthalate; MEOHP, mono(2-ethyl-5-oxohexyl) phthalate; MEP, monoethyl phthalate; MEPA, methyl paraben; MiBP, mono-iso-butyl phthalate; Mn, manganese; MnBP, mono-n-butyl phthalate; Mo, molybdenum; OHMiNP, mono-hydroxy-isononyl phthalate; OXBE, oxybenzone; OXOMiNP, mono-oxo-isononyl phthalate; Pb, lead; PBDEs, polybrominated diphenyl ethers; PCB, polychlorinated biphenyl; PFHxS, perfluorohexane sulfonate; PFNA, perfluorononanoic acid; PFOA, perfluoro-octanoic acid; PFOS, perfluoro-octane sulfonate; PFUnDA, perfluoroundecanoic acid; PRPA, propyl paraben; and TCS, triclosan	

© 2024 Güil-Oumrait N et al. *JAMA Network Open.*

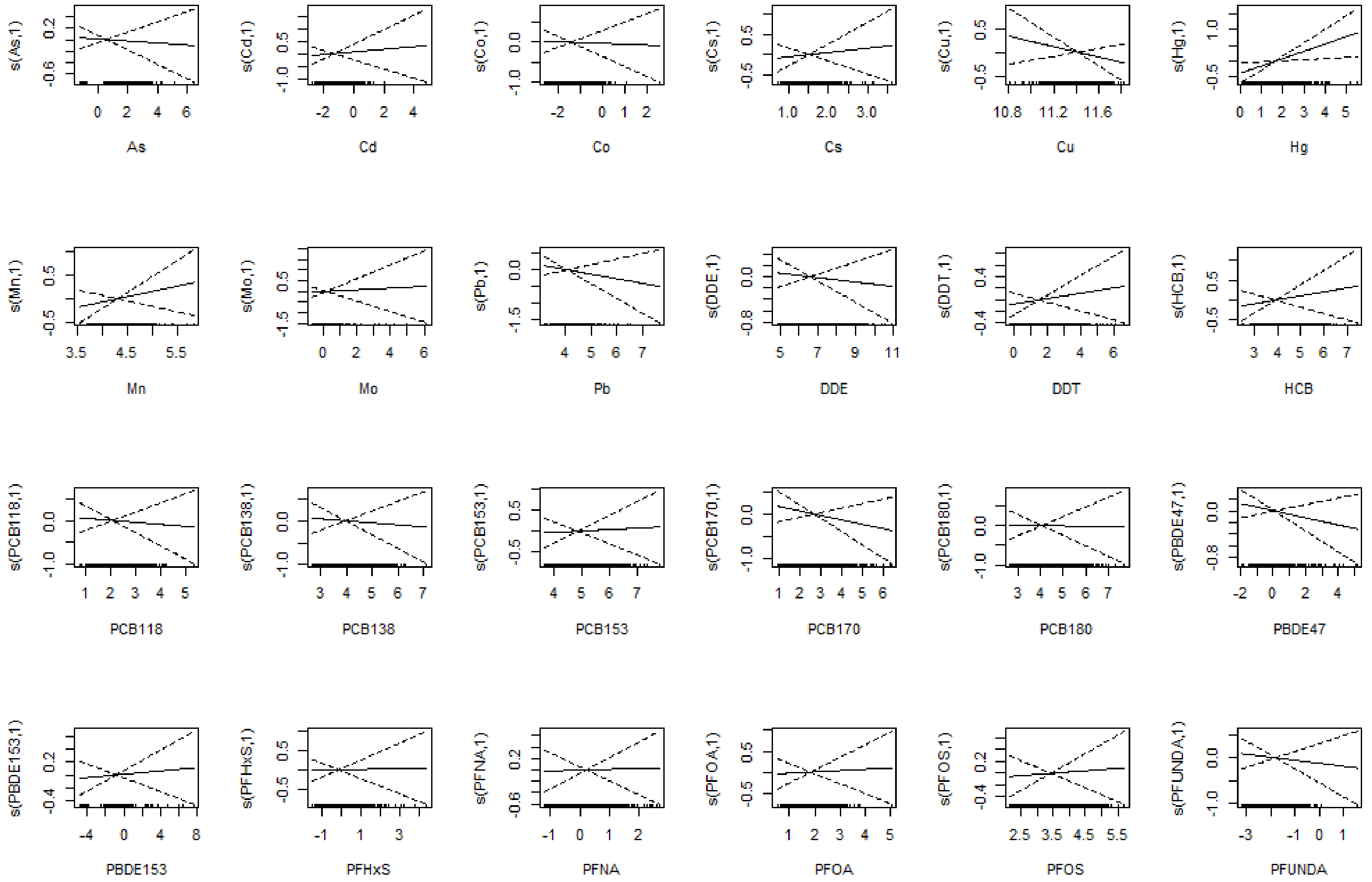

eFigure 2. Generalized Additive Models of Prenatal Exposure to Metals and Persistent Chemicals and Child Metabolic Syndrome Risk Score to Test Linearity Prior Using BWQS

Most of the associations were linear, strengthening the rationale for opting for BWQS as the main mixture method. All models were adjusted for subcohort, parental country of birth, maternal age, maternal education level, maternal pre-pregnancy body mass index, parity, maternal smoking in pregnancy, and maternal fish intake in pregnancy. Abbreviations: As, inorganic arsenic; Cd, cadmium; Co, cobalt; Cs, caesium; Cu, copper; DDE, dichlorodiphenyldichloroethylene; DDT, dichlorodiphenyltrichloroethane; HCB, hexachlorobenzene; Hg, mercury; Mn, manganese; Mo, molybdenum; Pb, lead; PBDE, polybrominated diphenyl ether; PCB, polychlorinated biphenyl; PFHxS, perfluorohexane sulfonate; PFNA, perfluorononanoic acid; PFOA, perfluoro-octanoic acid; PFOS, perfluoro-octane sulfonate; and PFUnDA, perfluoroundecanoic acid.

© 2024 Güil-Oumrait N et al. *JAMA Network Open.*

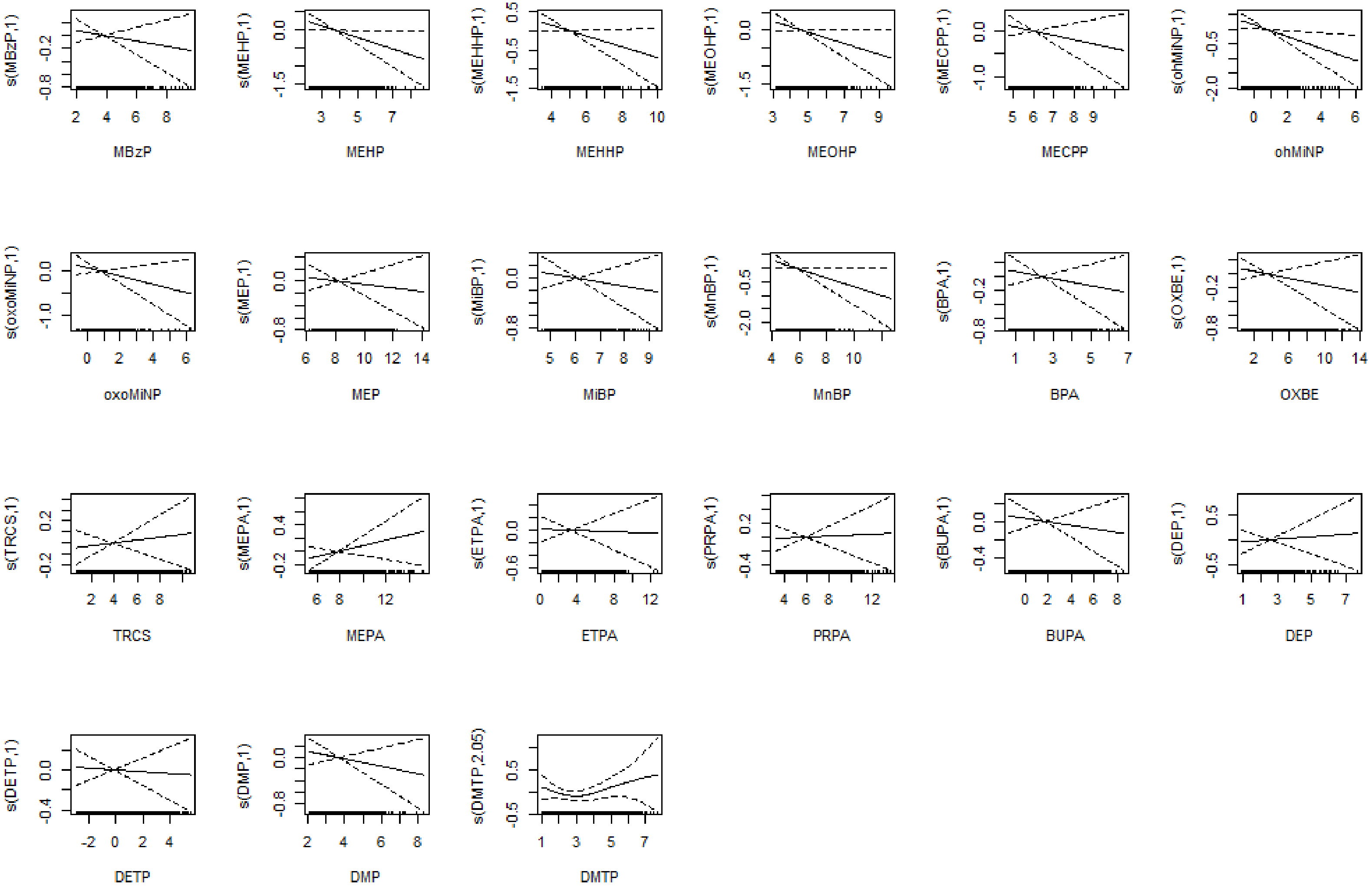

eFigure 3. Generalized Additive Models of Prenatal Exposure to Nonpersistent Chemicals and Child Metabolic Syndrome Risk Score to Test Linearity Prior Using BWQS

Most of the associations were linear, strengthening the rationale for opting for BWQS as the main mixture method. All models were adjusted for subcohort, parental country of birth, maternal age, maternal education level, maternal pre-pregnancy body mass index, parity, maternal smoking in pregnancy, and maternal fish intake in pregnancy. Abbreviations: BPA, bisphenol A; BUPA, N-butyl paraben; DEP, diethyl phosphate; DETP, diethylthiophosphate; DMP, dimethyl phosphate; DMTP, dimethylthiophosphate; ETPA, ethyl paraben; MBzP, monobenzylphthalate; MECPP,mono(2-ethyl-5-carboxypentyl) phthalate; MEHHP, mono(2-ethyl-5-hydroxyhexyl) phthalate; MEHP, mono-2-ethylhexyl phthalate; MEOHP, mono(2-ethyl-5-oxohexyl) phthalate; MEP, monoethyl phthalate; MEPA, methyl paraben; MiBP, mono-iso-butyl phthalate; MnBP, mono-n-butyl phthalate; OHMiNP, mono-hydroxy-isononyl phthalate; OXBE, oxybenzone; OXOMiNP, mono-oxo-isononyl phthalate; PRPA, propyl paraben; and TCS, triclosan.

© 2024 Güil-Oumrait N et al. *JAMA Network Open.*

eAppendix 2. Bayesian Weighted Quantile Sum (BWQS) Regression Characteristics

Hamiltonian Monte Carlo algorithm chain was set with a length of 10000 iterations. The scale-reduction statistic (R) of the Hamiltonian chain of all the models were approximately equal to 1, indicating optimal convergence[7]. Mixtures were set to be ranked in quartiles.

© 2024 Güil-Oumrait N et al. *JAMA Network Open.*

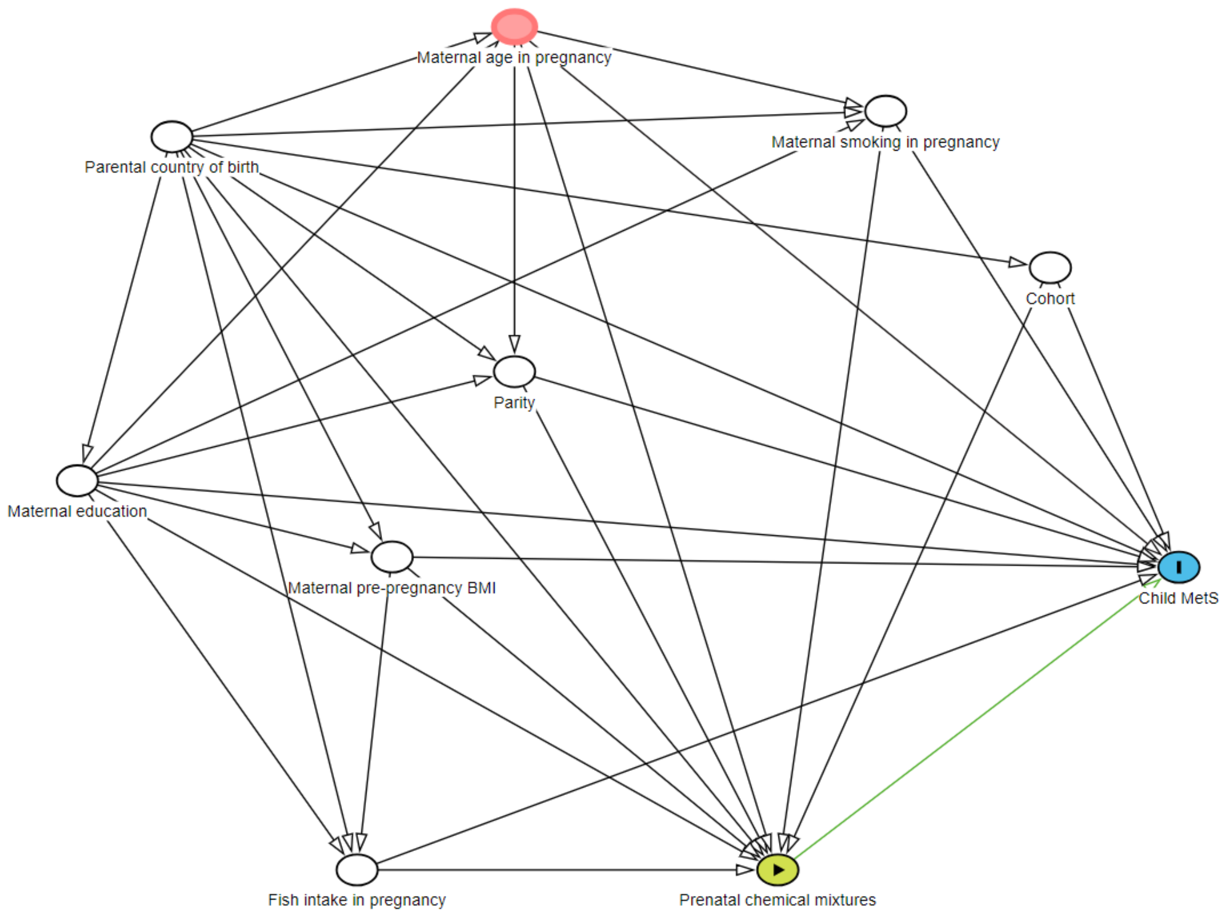

eFigure 4. Directed Acyclic Graph (DAG) Elucidating Exposure-Outcome Association

Red and white nodes: variables associated to both exposure and outcomes (confounders). Including only those variables with the white nodes, the model is considered to be sufficiently adjusted for the estimation of the total effect (direct and indirect) between prenatal chemical mixtures and child MetS risk score.

Abbreviations: BMI, body mass index; and MetS, metabolic syndrome.

© 2024 Güil-Oumrait N et al. *JAMA Network Open.*

eTable 4. Confounder-Only Model for MetS Score (N=1134)

Adjustment factors in the model	MetS score β (95%)	p-value
Cohort		
EDEN, France (vs. BIB)	-0.21 (-1.53, 1.12)	0.76
INMA, Spain (vs. BIB)	0.52 (-0.31, 1.34)	0.22
KANC, Lithuania (vs. BIB)	-0.77 (-1.30, -0.24)	0.004
MOBA, Norway (vs. BIB)	-1.04 (-1.80, -0.27)	0.008
RHEA, Greece	-0.46 (-1.03, 0.11)	0.11
Parental country of origin		
One or both parents native (vs. None native parent)	-0.12 (-0.60, 0.36)	0.60
Maternal age (years)	0.02 (-0.01, 0.05)	0.13
Maternal education		
Middle (vs. Low)	0.29 (-0.17, 0.75)	0.21
High (vs. Low)	0.09 (-0.37, 0.55)	0.69
Maternal pre-pregnancy BMI (kg/m^2)	0.09 (0.06, 0.12)	0.000
Parity		
Primiparous (vs. Nulliparous)	-0.08 (-0.38, 0.22)	0.59
Multiparous (vs. Nulliparous)	-0.08 (-0.48, 0.32)	0.70
Maternal fish intake in pregnancy		
Middle (vs. Low)	-0.08 (-0.48, 0.31)	0.68
High (vs. Low)	0.15 (-0.28, 0.59)	0.48
Maternal smoking in pregnancy: Yes (vs. No)	0.14 (-0.25, 0.53)	0.48
Child sex: Male (vs. Female)	-0.10 (-0.36, 0.16)	0.44
Child age at outcome assessment (years)	-0.02 (-0.31, 0.26)	0.88

Abbreviations: BiB, Born in Bradford; BMI, body mass index (calculated as weight in kilograms divided by height in meters squared); EDEN, Étude des Déterminants Pré et Postnatals du Développement et de la Santé de l'Enfant; INMA, Infancia y Medio Ambiente; KANC, Kaunas cohort; MoBa, Norwegian Mother, Father and Child cohort study; RHEA, RHEA Mother Child Cohort

© 2024 Güil-Oumrait N et al. *JAMA Network Open.*

eTable 5. Summary Statistics of Log_2-Transformed Prenatal EDC Concentrations

Chemical group	Units	Individual exposures	P25	P50	P75
Metals	µg/L	As	-0.85	0.66	1.43
		Cd	-1.75	-1.40	-1.04
		Co	-1.81	-1.52	-1.14
		Cs	1.24	1.46	1.73
		Cu	11.32	11.41	11.51
		Hg	1.00	1.54	2.19
		Mn	4.06	4.27	4.50
		Mo	-0.06	0.14	0.35
		Pb	3.78	4.04	4.32
OC Pesticides	ng/g of lipid	DDE	5.81	6.46	7.25
		DDT	0.72	1.09	2.09
		HCB	3.45	3.77	4.14
PCBs	ng/g of lipid	PCB-118	1.56	1.89	2.50
		PCB-138	3.49	3.87	4.37
		PCB-153	4.32	4.72	5.26
		PCB-170	2.01	2.58	3.22
		PCB-180	3.49	3.92	4.05
PBDEs	ng/g of lipid	PBDE-47	-0.77	-0.32	0.62
		PBDE-153	-1.35	-0.67	-0.11
PFASs	µg/L	PFHxS	-0.71	-0.26	0.25
		PFNA	-0.23	0.19	0.64
		PFOA	1.45	1.85	2.23
		PFOS	3.02	3.36	3.73
		PFUNDA	-2.22	-1.76	-1.33
HMWPs	µg/g of creatinine	MBzP	2.83	3.48	4.38
		MEHP	3.03	3.54	4.19

© 2024 Güil-Oumrait N et al. *JAMA Network Open.*

		MEHHP	4.36	4.82	5.38
		MEOHP	4.07	4.50	5.00
		MECPP	5.52	5.87	6.29
		oh-MiNP	0.27	0.60	1.11
		oxo-MiNP	0.24	0.70	1.25
LMWPs	µg/g of creatinine	MEP	7.14	7.94	9.07
		MiBP	5.54	5.97	6.42
		MnBP	5.19	5.56	6.04
Phenols	µg/g of creatinine	BPA	1.59	2.08	2.87
		OXBE	1.50	2.49	4.73
		TRCS	1.52	2.78	5.96
Parabens	µg/g of creatinine	MEPA	6.28	7.69	8.84
		ETPA	1.16	2.95	5.00
		PRPA	4.14	5.74	7.21
		BUPA	-0.70	1.51	3.65
OP Pesticide metabolites	µg/g of creatinine	DEP	1.91	2.40	3.05
		DETP	-2.00	-0.17	1.32
		DMP	3.04	3.60	4.28
		DMTP	2.05	2.83	3.18

Abbreviations: As, inorganic arsenic; BPA, bisphenol A; BUPA, N-butyl paraben; Cd, cadmium; Co, cobalt; Cs, caesium; Cu, copper; DDE, dichlorodiphenyldichloroethylene; DDT, dichlorodiphenyltrichloroethane; DEP, diethyl phosphate; DETP, diethylthiophosphate; DMP, dimethyl phosphate; DMTP, dimethylthiophosphate; ETPA, ethyl paraben; HCB, hexachlorobenzene; Hg, mercury; HMWPs, high-molecular-weight phthalate metabolites; LMWPs, low-molecular-weight phthalate metabolites; MBzP, monobenzylphthalate; MECPP,mono(2-ethyl-5-carboxypentyl) phthalate; MEHHP, mono(2-ethyl-5-hydroxyhexyl) phthalate; MEHP, mono-2-ethylhexyl phthalate; MEOHP, mono(2-ethyl-5-oxohexyl) phthalate; MEP, monoethyl phthalate; MEPA, methyl paraben; MiBP, mono-iso-butyl phthalate; Mn, manganese; MnBP, mono-n-butyl phthalate; Mo, molybdenum; OC, organochlorine; OHMiNP, mono-hydroxy-isononyl phthalate; OP, organophosphate; OXBE, oxybenzone; OXOMiNP, mono-oxo-isononyl phthalate; Pb, lead; PBDEs, polybrominated diphenyl ethers; PCBs, polychlorinated biphenyls; PFASs, perfluoroalkyl substances; PFHxS, perfluorohexane sulfonate; PFNA, perfluorononanoic acid; PFOA, perfluoro-octanoic acid; PFOS, perfluoro-octane sulfonate; PFUnDA, perfluoroundecanoic acid; PRPA, propyl paraben; and TCS, triclosan

© 2024 Güil-Oumrait N et al. *JAMA Network Open.*

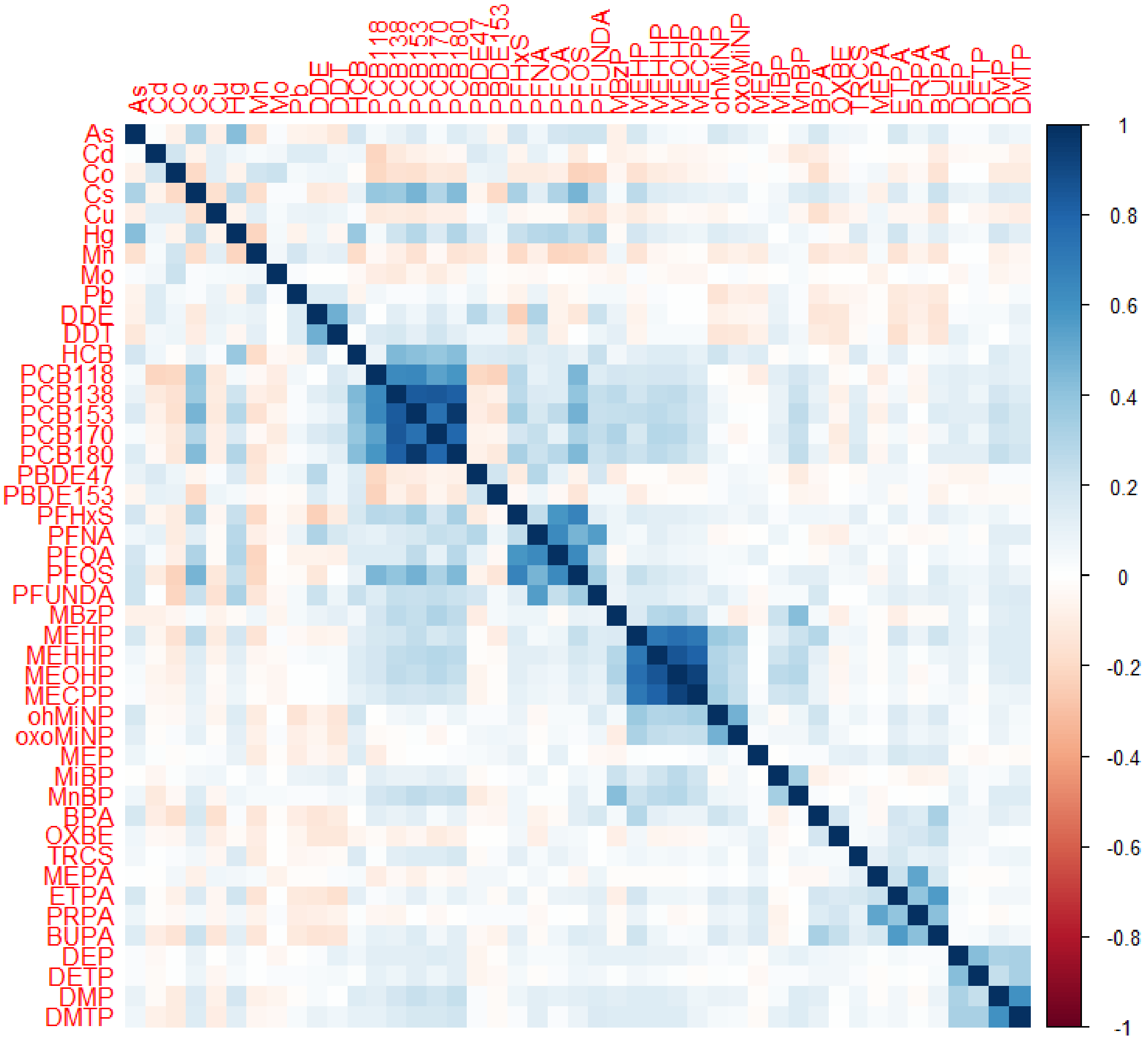

eFigure 5. Correlation Plot Between EDC Exposures

Abbreviations: As, inorganic arsenic; BPA, bisphenol A; BUPA, N-butyl paraben; Cd, cadmium; Co, cobalt; Cs, caesium; Cu, copper; DDE, dichlorodiphenyldichloroethylene; DDT, dichlorodiphenyltrichloroethane; DEP, diethyl phosphate; DETP, diethylthiophosphate; DMP, dimethyl phosphate; DMTP, dimethylthiophosphate; EDC, endocrine disrupting chemicals; ETPA, ethyl paraben; HCB, hexachlorobenzene; Hg, mercury; MBzP, monobenzylphthalate; MECPP,mono(2-ethyl-5-carboxypentyl) phthalate; MEHHP, mono(2-ethyl-5-hydroxyhexyl) phthalate; MEHP, mono-2-ethylhexyl phthalate; MEOHP, mono(2-ethyl-5-oxohexyl) phthalate; MEP, monoethyl phthalate; MEPA, methyl paraben; MiBP, mono-iso-butyl phthalate; Mn, manganese; MnBP, mono-n-butyl phthalate; Mo, molybdenum; OHMiNP, mono-hydroxy-isononyl phthalate; OP, organophosphate; OXBE, oxybenzone; OXOMiNP, mono-oxo-isononyl phthalate; Pb, lead; PBDEs, polybrominated diphenyl ether; PCB, polychlorinated biphenyl; PFHxS, perfluorohexane sulfonate; PFNA, perfluorononanoic acid; PFOA, perfluoro-octanoic acid; PFOS, perfluoro-octane sulfonate; PFUnDA, perfluoroundecanoic acid; PRPA, propyl paraben; and TCS, triclosan.

© 2024 Güil-Oumrait N et al. *JAMA Network Open.*

eTable 6. BWQS Models for MetS for All the Study Population and Stratified by Sex

Exposure mixture group	MetS score β (95% CrI) All (n=1134)	Girls (n= 517)	Boys (n = 617)
Metals	0.44 (0.30, 0.59)	0.53 (0.34, 0.72)	0.30 (0.05, 0.55)
OC pesticides	0.22 (0.15, 0.29)	0.29 (0.19, 0.38)	0.22 (0.15, 0.29)
PCBs	-0.05 (-0.09, 0.00)	0.11 (0.03, 0.19)	-0.17 (-0.21, -0.12)
PBDEs	0.17 (0.06, 0.27)	0.20 (0.06, 0.35)	0.16 (-0.02, 0.33)
PFASs	0.19 (0.14, 0.24)	0.29 (0.22, 0.36)	0.01 (-0.07, 0.10)
LMWPs	-0.13 (-0.18, -0.08)	-0.07 (-0.14, -0.01)	-0.15 (-0.25, -0.06)
HMWPs	-0.07 (-0.10, -0.04)	-0.16 (-0.24, -0.08)	0.01 (-0.05, 0.06)
Phenols	-0.06 (-0.15, 0.04)	-0.16 (-0.34, 0.01)	0.02 (-0.06, 0.10)
Parabens	0.06 (-0.02, 0.13)	-0.02 (-0.12, 0.08)	0.11 (0.03, 0.19)
OP Pesticide metabolites	0.00 (-0.04, 0.04)	0.04 (-0.02, 0.10)	-0.03 (-0.10, 0.04)

All models were adjusted for subcohort, parental country of birth, maternal age, maternal education level, maternal pre-pregnancy body mass index, parity, maternal smoking in pregnancy, and maternal fish intake in pregnancy. Abbreviations: BWQS, Bayesian weighted quantile sum; CrI, credible interval; HMWPs, high-molecular-weight phthalate metabolites; LMWPs, low-molecular-weight phthalate metabolites; MetS, Metabolic Syndrome; OC, organochlorine; OP, organophosphate; PBDEs, polybrominated diphenyl ethers; PCBs, polychlorinated biphenyls; PFASs, perfluoroalkyl substances.

© 2024 Güil-Oumrait N et al. *JAMA Network Open.*

eTable 7. Estimated Posterior Weights of Exposure Mixture Groups on MetS Using BWQS Models for All the Study Population and Stratified by Sex

		MetS score		
**Exposure group**	**Exposure variable**	**All (n=1134)**	**Girls (n= 517)**	**Boys (n= 617)**
			**Weight (95% CrI)**	
Metals	As	0.08 (0.02, 0.15)	0.10 (0.03, 0.16)	0.10 (0.07, 0.14)
	Cd	0.14 (0.07, 0.21)	0.15 (0.06, 0.23)	0.12 (0.07, 0.16)
	Co	0.09 (0.04, 0.14)	0.08 (0.04, 0.12)	0.12 (0.06, 0.18)
	Cs	0.06 (0.04, 0.09)	0.09 (0.05, 0.14)	0.08 (0.06, 0.11)
	Cu	0.06 (0.03, 0.10)	0.09 (0.05, 0.13)	0.09 (0.05, 0.12)
	Hg	0.33 (0.21, 0.45)	0.25 (0.13, 0.37)	0.18 (0.12, 0.24)
	Mn	0.06 (0.03, 0.09)	0.07 (0.04, 0.09)	0.09 (0.05, 0.13)
	Mo	0.08 (0.04, 0.13)	0.08 (0.05, 0.11)	0.11 (0.05, 0.17)
	Pb	0.08 (0.05, 0.12)	0.09 (0.04, 0.13)	0.10 (0.08, 0.13)
OC pesticides	DDE	0.27 (0.15, 0.38)	0.26 (0.16, 0.36)	0.27 (0.15, 0.38)
	DDT	0.22 (0.08, 0.36)	0.26 (0.11, 0.41)	0.22 (0.08, 0.36)
	HCB	0.51 (0.37, 0.66)	0.48 (0.35, 0.62)	0.51 (0.37, 0.66)
PCBs	PCB118	0.22 (0.18, 0.26)	0.17 (0.14, 0.21)	0.22 (0.17, 0.27)
	PCB138	0.18 (0.16, 0.21)	0.20 (0.17, 0.24)	0.15 (0.12, 0.18)
	PCB153	0.20 (0.19, 0.22)	0.19 (0.16, 0.22)	0.21 (0.18, 0.25)
	PCB170	0.20 (0.17, 0.23)	0.23 (0.16, 0.30)	0.22 (0.16, 0.28)
	PCB180	0.19 (0.17, 0.21)	0.21 (0.17, 0.24)	0.19 (0.16, 0.23)
PBDEs	PBDE47	0.53 (0.24, 0.82)	0.47 (0.20, 0.73)	0.57 (0.31, 0.84)
	PBDE153	0.47 (0.18, 0.76)	0.53 (0.27, 0.80)	0.43 (0.16, 0.69)
PFASs	PFHxS	0.13 (0.10, 0.15)	0.16 (0.13, 0.20)	0.19 (0.17, 0.21)
	PFNA	0.48 (0.41, 0.52)	0.40 (0.32, 0.48)	0.23 (0.17, 0.28)
	PFOA	0.16 (0.13, 0.18)	0.17 (0.15, 0.20)	0.19 (0.17, 0.20)
	PFOS	0.10 (0.07, 0.12)	0.12 (0.09, 0.15)	0.20 (0.16, 0.24)
	PFUNDA	0.14 (0.10, 0.19)	0.14 (0.10, 0.19)	0.20 (0.18, 0.21)
LMWPs	MEP	0.23 (0.18, 0.28)	0.30 (0.24, 0.35)	0.31 (0.22, 0.39)
	MiBP	0.29 (0.21, 0.38)	0.31 (0.26, 0.36)	0.29 (0.21, 0.36)
	MnBP	0.48 (0.37, 0.58)	0.39 (0.31, 0.47)	0.41 (0.30, 0.51)
HMWPs	MBzP	0.14 (0.12, 0.15)	0.12 (0.10, 0.14)	0.15 (0.13, 0.16)
	MEHP	0.16 (0.14, 0.17)	0.16 (0.14, 0.18)	0.14 (0.13, 0.15)
	MEHHP	0.15 (0.13, 0.18)	0.15 (0.12, 0.17)	0.14 (0.13, 0.15)
	MEOHP	0.14 (0.13, 0.16)	0.14 (0.12, 0.16)	0.14 (0.13, 0.15)
	MECPP	0.14 (0.12, 0.15)	0.14 (0.12, 0.16)	0.14 (0.13, 0.15)
	oh-MiNP	0.13 (0.12, 0.15)	0.14 (0.11, 0.18)	0.15 (0.13, 0.17)
	oxo-MiNP	0.14 (0.12, 0.17)	0.15 (0.12, 0.18)	0.15 (0.14, 0.16)
Phenols	BPA	0.36 (0.26, 0.46)	0.37 (0.28, 0.47)	0.33 (0.29, 0.38)
	OXBE	0.35 (0.28, 0.43)	0.40 (0.30, 0.50)	0.33 (0.31, 0.36)
	TRCS	0.28 (0.16, 0.40)	0.23 (0.09, 0.37)	0.33 (0.29, 0.38)
Parabens	MEPA	0.26 (0.21, 0.31)	0.24 (0.22, 0.27)	0.30 (0.22, 0.37)
	ETPA	0.28 (0.22, 0.33)	0.25 (0.21, 0.29)	0.25 (0.22, 0.28)
	PRPA	0.24 (0.20, 0.28)	0.25 (0.22, 0.27)	0.24 (0.20, 0.28)
	BUPA	0.22 (0.18, 0.27)	0.26 (0.22, 0.30)	0.21 (0.16, 0.26)

© 2024 Güil-Oumrait N et al. *JAMA Network Open.*

OP Pesticide metabolites	DEP	0.25 (0.23, 0.26)	0.25 (0.23, 0.27)	0.25 (0.22, 0.29)
	DETP	0.25 (0.22, 0.28)	0.25 (0.23, 0.27)	0.27 (0.21, 0.33)
	DMP	0.25 (0.22, 0.28)	0.26 (0.23, 0.29)	0.24 (0.20, 0.27)
	DMTP	0.25 (0.23, 0.27)	0.24 (0.22, 0.26)	0.24 (0.20, 0.28)

All models were adjusted for subcohort, parental country of birth, maternal age, maternal education level, maternal pre-pregnancy body mass index, parity, maternal smoking in pregnancy, and maternal fish intake in pregnancy. Abbreviations: As, inorganic arsenic; BPA, bisphenol A; BUPA, N-butyl paraben; Cd, cadmium; Co, cobalt; CrI, credible interval; Cs, caesium; Cu, copper; DDE, dichlorodiphenyldichloroethylene; DDT, dichlorodiphenyltrichloroethane; DEP, diethyl phosphate; DETP, diethylthiophosphate; DMP, dimethyl phosphate; DMTP, dimethylthiophosphate; ETPA, ethyl paraben; HCB, hexachlorobenzene; Hg, mercury; HMWPs, high-molecular-weight phthalate metabolites; LMWPs, low-molecular-weight phthalate metabolites; MBzP, monobenzylphthalate; MECPP,mono(2-ethyl-5-carboxypentyl) phthalate; MEHHP, mono(2-ethyl-5-hydroxyhexyl) phthalate; MEHP, mono-2-ethylhexyl phthalate; MEOHP, mono(2-ethyl-5-oxohexyl) phthalate; MEP, monoethyl phthalate; MEPA, methyl paraben; MetS, Metabolic Syndrome; MiBP, mono-iso-butyl phthalate; Mn, manganese; MnBP, mono-n-butyl phthalate; Mo, molybdenum; OC, organochlorine; OHMiNP, mono-hydroxy-isononyl phthalate; OP, organophosphate; OXBE, oxybenzone; OXOMiNP, mono-oxo-isononyl phthalate; Pb, lead; PBDEs, polybrominated diphenyl ethers; PCBs, polychlorinated biphenyls; PFASs, perfluoroalkyl substances; PFHxS, perfluorohexane sulfonate; PFNA, perfluorononanoic acid; PFOA, perfluoro-octanoic acid; PFOS, perfluoro-octane sulfonate; PFUnDA, perfluoroundecanoic acid; PRPA, propyl paraben; and TCS, triclosan

© 2024 Güil-Oumrait N et al. *JAMA Network Open.*

eTable 8. Adjusted Associations Between Childhood Proteins and Child MetS Risk Score

Child proteins	β	SE	p-value	FDR adjusted p-value
C-reactive protein	0.240	0.038	0.000	0.000
APO-A1	-1.043	0.145	0.000	0.000
APO-E	0.520	0.109	0.000	0.000
IL1 beta	1.453	0.064	0.000	0.000
IL6	1.747	0.087	0.000	0.000
Leptin	0.443	0.054	0.000	0.000
HGF	2.360	0.256	0.000	0.000
TNF-alpha	1.438	0.211	0.000	0.000
B-cell activating factor	2.142	0.233	0.000	0.000
C-peptide	0.165	0.027	0.000	0.000
IL8	1.400	0.203	0.000	0.000
IL1RA	0.335	0.068	0.000	0.000
MCP1	0.508	0.164	0.002	0.005
Adiponectin	-0.401	0.133	0.003	0.006
IL10	0.088	0.048	0.068	0.114
IL13	0.104	0.073	0.152	0.238
PAI1	0.091	0.066	0.165	0.247
IL5	0.074	0.055	0.176	0.261
IP10	0.106	0.082	0.194	0.283
IL12	-0.131	0.112	0.239	0.330
APO-B	0.222	0.189	0.242	0.332
IL2	0.053	0.054	0.327	0.431
MIP1 beta	0.099	0.103	0.335	0.434
FGF basic	-0.026	0.037	0.476	0.572
Eotaxin	-0.085	0.122	0.485	0.578
IL15	0.020	0.038	0.601	0.689
EGF	-0.016	0.035	0.641	0.716
MIG	0.041	0.091	0.651	0.724
IL2 receptor	-0.039	0.090	0.665	0.736
IFN gamma	-0.020	0.049	0.686	0.745
IL4	0.029	0.073	0.689	0.745
IFN alfa	0.026	0.069	0.710	0.760
GCSF	0.018	0.112	0.873	0.898
MIP1alfa	0.015	0.108	0.890	0.911
IL17	-0.004	0.053	0.939	0.944

Effect estimates were expressed per doubling of child protein levels. All models were adjusted for subcohort, parental country of birth, maternal age, maternal education level, maternal pre-pregnancy body mass index, parity, maternal smoking in pregnancy, and maternal fish intake in pregnancy. Abbreviations: APO, apolipoprotein; EGF, Epidermal growth factor; FDR, false discovery rate; FGF, fibroblast growth factor; GCSF, granulocyte-colony stimulating factor; HGF, hepatocyte growth factor; IFN, interferon; IL, interleukin; IL1RA, interleukin 1 receptor antagonist; IP10, Interferon gamma-induced protein 10; MCP1, Monocyte Chemoattractant Protein-1; MetS, Metabolic Syndrome; MIG, monokine induced by gamma interferon; MIP1 beta, Macrophage inflammatory protein; PAI1, Plasminogen activator inhibitor-1; SE, standard error; TNF, Tumour Necrosis Factor

© 2024 Güil-Oumrait N et al. *JAMA Network Open.*

eTable 9. Adjusted Associations Between Childhood Serum Metabolites and Child MetS Risk Score

Child serum metabolites	β	SE	p-value	FDR-adjusted p-value
Alanine	2.503	0.248	0.000	0.000
C0	2.133	0.276	0.000	0.000
C10	-1.318	0.183	0.000	0.000
C10.1	-0.931	0.174	0.000	0.000
C12	-1.533	0.215	0.000	0.000
C12.1	-1.723	0.218	0.000	0.000
C14	-1.175	0.28	0.000	0.000
C14.1	-0.899	0.111	0.000	0.000
C14.1.OH	-1.323	0.294	0.000	0.000
C14.2	-1.029	0.134	0.000	0.000
C16	-1.005	0.233	0.000	0.000
C16.1	-1.423	0.246	0.000	0.000
C16.2	-1.064	0.279	0.000	0.000
C18	-0.99	0.25	0.000	0.000
C18.1	-1.167	0.219	0.000	0.000
C2	-1.087	0.137	0.000	0.000
C3	1.166	0.157	0.000	0.000
C4	0.641	0.145	0.000	0.000
C5	0.981	0.183	0.000	0.000
C7.DC	-1.576	0.279	0.000	0.000
C8	-1.742	0.256	0.000	0.000
Glutamate	1.176	0.162	0.000	0.000
H1	4.53	0.527	0.000	0.000
Isoleucine	0.897	0.173	0.000	0.000
Kynurenine	1.412	0.255	0.000	0.000
Leucine	0.975	0.188	0.000	0.000
Lysine	0.933	0.206	0.000	0.000
lysoPC.a.C14.0	3.293	0.404	0.000	0.000
lysoPC.a.C16.0	1.354	0.248	0.000	0.000
lysoPC.a.C16.1	1.622	0.184	0.000	0.000
lysoPC.a.C18.0	0.949	0.22	0.000	0.000
lysoPC.a.C20.3	0.963	0.16	0.000	0.000
Methionine sulfoxide	0.567	0.144	0.000	0.000
Ornithine	0.871	0.221	0.000	0.000
PC.aa.C32.1	0.827	0.142	0.000	0.000
PC.aa.C34.3	1.007	0.196	0.000	0.000
PC.aa.C34.4	0.794	0.149	0.000	0.000
PC.aa.C36.0	-1.001	0.218	0.000	0.000
PC.aa.C36.5	0.536	0.128	0.000	0.000
PC.aa.C38.0	-1.003	0.216	0.000	0.000
PC.aa.C38.3	1.708	0.227	0.000	0.000
PC.aa.C42.0	-1.013	0.259	0.000	0.000
PC.ae.C30.2	0.785	0.203	0.000	0.000

© 2024 Güil-Oumrait N et al. *JAMA Network Open.*

PC.ae.C32.1	-1.61	0.256	0.000	0.000
PC.ae.C34.1	-1.281	0.283	0.000	0.000
PC.ae.C34.2	-1.318	0.229	0.000	0.000
PC.ae.C34.3	-1.877	0.214	0.000	0.000
PC.ae.C36.0	-1.412	0.26	0.000	0.000
PC.ae.C36.3	-1.373	0.248	0.000	0.000
PC.ae.C36.4	-1.031	0.224	0.000	0.000
PC.ae.C36.5	-1.077	0.207	0.000	0.000
PC.ae.C38.4	-1.434	0.274	0.000	0.000
PC.ae.C38.5	-1.478	0.256	0.000	0.000
PC.ae.C38.6	-0.924	0.216	0.000	0.000
PC.ae.C40.4	-1.24	0.261	0.000	0.000
PC.ae.C40.5	-1.445	0.271	0.000	0.000
PC.ae.C40.6	-1.324	0.25	0.000	0.000
PC.ae.C42.4	-1.481	0.234	0.000	0.000
PC.ae.C42.5	-1.162	0.258	0.000	0.000
PC.ae.C44.4	-1.187	0.258	0.000	0.000
PC.ae.C44.5	-0.946	0.207	0.000	0.000
PC.ae.C44.6	-1.153	0.227	0.000	0.000
Phenilalanine	2.46	0.262	0.000	0.000
Proline	1.607	0.173	0.000	0.000
SM.C16.0	-2.111	0.35	0.000	0.000
SM.C20.2	-0.883	0.202	0.000	0.000
Tryptophan	1.525	0.256	0.000	0.000
Tyrosine	1.426	0.209	0.000	0.000
Valine	1.452	0.236	0.000	0.000
Asymmetric dimethylarginine	1.506	0.405	0.000	0.001
Arginine	1.029	0.284	0.000	0.001
Methionine	0.648	0.181	0.000	0.001
PC.aa.C32.2	0.546	0.148	0.000	0.001
PC.aa.C36.6	0.561	0.15	0.000	0.001
PC.aa.C40.1	-1.286	0.371	0.001	0.001
PC.aa.C40.6	0.732	0.201	0.000	0.001
PC.ae.C36.2	-0.937	0.266	0.000	0.001
PC.aa.C36.1	0.77	0.232	0.001	0.002
PC.aa.C38.1	-0.493	0.148	0.001	0.002
SM.C24.1	-0.783	0.23	0.001	0.002
Alpha-AAA	0.352	0.107	0.001	0.003
lysoPC.a.C20.4	0.632	0.195	0.001	0.003
lysoPC.a.C28.0	0.541	0.17	0.002	0.004
PC.aa.C40.5	0.741	0.231	0.001	0.004
C18.2	-0.707	0.23	0.002	0.005
PC.ae.C42.3	-0.775	0.252	0.002	0.005
PC.aa.C38.4	0.741	0.245	0.003	0.006
SM..OH..C16.1	-0.762	0.249	0.002	0.006
C3.OH	0.633	0.212	0.003	0.007

© 2024 Güil-Oumrait N et al. *JAMA Network Open.*

PC.ae.C32.2	-0.769	0.257	0.003	0.007
lysoPC.a.C28.1	0.524	0.181	0.004	0.009
PC.aa.C32.0	-0.841	0.291	0.004	0.009
PC.aa.C24.0	0.356	0.127	0.005	0.011
SM.C26.0	-0.458	0.163	0.005	0.011
PC.aa.C42.1	-0.706	0.253	0.005	0.012
Histidine	1.088	0.401	0.007	0.014
SM.C26.1	-0.473	0.174	0.007	0.014
lysoPC.a.C26.0	0.317	0.122	0.009	0.020
PC.aa.C40.4	0.672	0.258	0.009	0.020
C5.DC..C6.OH.	-0.823	0.321	0.010	0.022
Threonine	0.618	0.242	0.011	0.022
C16.1.OH	-0.773	0.31	0.013	0.026
PC.aa.C32.3	0.583	0.241	0.016	0.032
Symmetric dimethylarginine	-0.17	0.071	0.017	0.034
PC.ae.C34.0	-0.502	0.211	0.017	0.035
lysoPC.a.C17.0	0.467	0.201	0.021	0.040
PC.aa.C36.3	0.615	0.27	0.023	0.044
SM..OH..C24.1	-0.474	0.208	0.023	0.044
PC.aa.C30.0	0.406	0.18	0.024	0.046
Aspartate	0.482	0.216	0.026	0.049
SM.C18.0	-0.556	0.254	0.029	0.054
PC.aa.C42.2	-0.576	0.269	0.032	0.060
lysoPC.a.C26.1	0.256	0.122	0.037	0.067
C6..C4.1.DC.	-0.545	0.264	0.039	0.072
C9	-0.653	0.318	0.040	0.073
Glutamine	-0.852	0.426	0.046	0.081
PC.ae.C40.2	-0.52	0.264	0.049	0.087
C5.1.DC	-0.575	0.296	0.052	0.091
SM..OH..C14.1	-0.453	0.242	0.062	0.108
Serotonin	-0.174	0.094	0.064	0.110
C3.DC..C4.OH.	-0.278	0.15	0.065	0.111
Serine	-0.635	0.345	0.066	0.112
C5.OH..C3.DC.M.	1.041	0.584	0.075	0.126
PC.aa.C42.4	-0.48	0.273	0.080	0.133
PC.aa.C40.2	-0.46	0.263	0.081	0.134
C16.2.OH	0.847	0.495	0.088	0.144
PC.aa.C36.4	0.452	0.269	0.094	0.153
C4.1	0.597	0.358	0.096	0.156
PC.ae.C42.2	-0.403	0.246	0.101	0.163
Citrulline	-0.373	0.232	0.108	0.174
Glycine	-0.436	0.3	0.146	0.231
Acetyl-Ornithine	-0.095	0.067	0.154	0.240
PC.aa.C28.1	0.352	0.248	0.155	0.240
C16.OH	0.321	0.231	0.165	0.247
PC.aa.C38.5	0.332	0.239	0.165	0.247

© 2024 Güil-Oumrait N et al. *JAMA Network Open.*

SM.C24.0	-0.348	0.249	0.162	0.247
PC.aa.C36.2	0.371	0.271	0.172	0.256
PC.aa.C40.3	-0.376	0.283	0.185	0.273
PC.ae.C42.1	0.344	0.265	0.195	0.283
PC.ae.C40.3	-0.313	0.242	0.196	0.284
PC.ae.C38.0	0.257	0.201	0.201	0.289
C12.DC	-0.917	0.729	0.209	0.296
PC.aa.C30.2	0.029	0.023	0.208	0.296
C5.M.DC	-0.392	0.325	0.228	0.321
Taurine	-0.238	0.197	0.228	0.321
PC.ae.C30.1	0.122	0.103	0.234	0.327
lysoPC.a.C18.2	0.172	0.147	0.244	0.333
PC.ae.C38.2	-0.273	0.239	0.253	0.344
lysoPC.a.C18.1	0.216	0.194	0.266	0.360
PC.ae.C42.0	0.566	0.56	0.312	0.415
SM..OH..C22.1	-0.234	0.234	0.317	0.420
PC.aa.C42.5	-0.26	0.266	0.329	0.431
lysoPC.a.C24.0	0.166	0.18	0.357	0.460
Putrescine	-0.078	0.089	0.379	0.482
PC.ae.C44.3	0.205	0.236	0.385	0.485
Total dimethylamine	-0.38	0.437	0.384	0.485
PC.aa.C34.2	-0.245	0.284	0.389	0.487
PC.ae.C40.1	0.192	0.233	0.410	0.507
SM..OH..C22.2	-0.188	0.235	0.424	0.521
PC.aa.C42.6	-0.232	0.292	0.427	0.523
C6.1	-0.282	0.382	0.460	0.558
PC.ae.C36.1	-0.187	0.253	0.461	0.558
PC.aa.C34.1	0.184	0.253	0.466	0.563
C10.2	0.26	0.385	0.499	0.591
PC.ae.C30.0	-0.113	0.218	0.603	0.689
SM.C18.1	-0.134	0.266	0.615	0.699
PC.aa.C38.6	-0.099	0.204	0.629	0.706
C14.2.OH	-0.137	0.333	0.682	0.745
PC.ae.C38.1	-0.029	0.074	0.689	0.745
PC.ae.C38.3	0.096	0.241	0.690	0.745
Spermine	-0.04	0.097	0.680	0.745
C5.1	0.145	0.383	0.706	0.759
Spermidine	0.082	0.309	0.791	0.833
t4.OH.Pro	-0.035	0.168	0.837	0.868
Aspargine	0.04	0.243	0.869	0.897
SM.C16.1	0.025	0.326	0.940	0.944
Creatinine	0.022	0.338	0.947	0.947

Effect estimates were expressed per doubling of child serum metabolite levels. All models were adjusted for subcohort, parental country of birth, maternal age, maternal education level, maternal pre-pregnancy body mass index, parity, maternal smoking in pregnancy, and maternal fish intake in pregnancy. Abbreviations: AAA, aminoadipic acid; FDR, false discovery rate; MetS, metabolic syndrome; PC, phosphatidylcoline; SE, standard error; SM, sphingomyelin.

© 2024 Güil-Oumrait N et al. *JAMA Network Open.*

eTable 10. Adjusted Associations Between Childhood Urine Metabolites and Child MetS Risk Score

Child urine metabolites	β	SE	p-value	FDR-adjusted p-value
Valine	1.005	0.23	0.000	0.000
X4.deoxyerythronic.acid	0.999	0.148	0.000	0.000
p.cresol.sulfate	-0.268	0.056	0.000	0.000
Tyrosine	0.487	0.118	0.000	0.000
Leucine	0.63	0.206	0.002	0.006
Acetone	-0.165	0.054	0.002	0.006
Hippurate	-0.2	0.071	0.005	0.011
Alanine	0.384	0.138	0.006	0.012
X3.aminoisobutyrate	-0.138	0.054	0.010	0.022
X3.hydroxyisobutyrate	0.352	0.148	0.017	0.035
Isoleucine	0.129	0.059	0.028	0.054
X3.Indoxylsulfate	-0.103	0.048	0.033	0.061
X4.deoxythreonic.acid	-0.245	0.121	0.043	0.077
Taurine	-0.087	0.043	0.045	0.081
Carnitine	-0.073	0.045	0.110	0.175
N.methylpicolinic.acid	0.041	0.027	0.123	0.195
X3.hydroxybutyrate.3.aminoisobutyrate	-0.051	0.036	0.158	0.243
Acetate	-0.115	0.088	0.189	0.278
Pantothenic.acid	-0.222	0.188	0.236	0.328
Scyllo.inositol	0.058	0.053	0.275	0.370
N.methylnicotinic.acid	0.066	0.062	0.288	0.386
Citrate	0.085	0.087	0.331	0.432
X2.hydroxyisobutyrate	0.166	0.182	0.362	0.465
N.methyl.2.pyridone.5.carboxamide	0.028	0.032	0.374	0.478
Glutamine	0.079	0.094	0.399	0.498
Formate	0.081	0.097	0.403	0.500
Proline.betaine	0.014	0.02	0.486	0.578
Urea	-0.123	0.186	0.508	0.599
X5.oxoproline	-0.047	0.073	0.515	0.605
Creatine	0.028	0.046	0.545	0.637
Dimethylamine	0.048	0.082	0.561	0.653
Lactate	-0.08	0.145	0.579	0.667
Trimethylamine	-0.061	0.109	0.576	0.667
Succinate	-0.018	0.035	0.619	0.700
Creatinine	-0.121	0.246	0.622	0.701
Lysine	0.032	0.095	0.732	0.777
Glycine	0.038	0.108	0.728	0.777
p.hydroxyphenylacetate	-0.041	0.128	0.747	0.790
X3.hydroxyisovalerate	-0.035	0.139	0.801	0.840
Sucrose	0.009	0.038	0.815	0.851
N1.methyl.nicotinamide	0.014	0.066	0.831	0.864
Glucose	0.014	0.109	0.899	0.917
N.acetyl.neuraminic.acid	0.008	0.067	0.906	0.920

© 2024 Güil-Oumrait N et al. *JAMA Network Open.*

Trimethylamine.oxide	0.004	0.043	0.924	0.934

Effect estimates were expressed per doubling of child urine metabolite levels. All models were adjusted for subcohort, parental country of birth, maternal age, maternal education level, maternal pre-pregnancy body mass index, parity, maternal smoking in pregnancy, and maternal fish intake in pregnancy. Abbreviations: FDR, false discovery rate; MetS, metabolic syndrome; SE, standard error

© 2024 Güil-Oumrait N et al. *JAMA Network Open.*

eTable 11. Adjusted Associations Between Prenatal EDC Mixtures and Child Proteins Using BWQS

	Metals mixture			OCs mixture			PBDEs mixture			PFASs mixture			LMWPs mixture			HMWPs mixture		
**Omics**	**β**	**LCrI**	**UCrI**	**β**	**LCrI**	**UCrI**	**β**	**LCrI**	**UCrI**	**β**	**LCrI**	**UCrI**	**β**	**LCrI**	**UCrI**	**β**	**LCrI**	**UCrI**
**Adiponectin**	-0,40	2,63	-3,33	-0,02	1,16	-1,20	-0,74	2,27	-3,66	-0,89	-0,01	-1,76	**-1,30**	**-0,06**	**-2,53**	0,16	1,33	-0,98
**APO.A1**	**5,17**	**7,55**	**2,84**	**4,51**	**5,57**	**3,45**	-1,32	-0,07	-2,56	**3,34**	**4,20**	**2,48**	**4,35**	**5,25**	**3,47**	**4,94**	**5,60**	**4,29**
**APO.E**	**4,48**	**7,17**	**1,87**	**6,71**	**8,15**	**5,28**	**1,88**	**3,25**	**0,54**	**1,95**	**2,92**	**0,99**	**2,35**	**3,89**	**0,83**	0,70	1,95	-0,54
**BAFF**	2,06	4,49	-0,32	**-0,84**	**-0,11**	**-1,56**	**1,82**	**2,52**	**1,11**	**-1,12**	**-0,75**	**-1,49**	**-1,62**	**-0,84**	**-2,40**	**-0,87**	**-0,30**	**-1,43**
**Cpeptide**	3,67	12,18	-4,19	**-11,84**	**-7,70**	**-15,80**	0,55	10,98	-8,90	**5,40**	**8,44**	**2,44**	6,80	15,60	-1,32	-1,40	1,83	-4,51
**CRP**	**30,25**	**40,35**	**20,87**	**22,35**	**28,14**	**16,82**	**9,29**	**15,52**	**3,40**	**10,10**	**13,38**	**6,92**	**6,79**	**11,45**	**2,32**	**11,34**	**15,02**	**7,77**
**HGF**	1,24	4,25	-1,69	0,00	0,90	-0,90	**1,76**	**2,60**	**0,92**	**-0,68**	**-0,21**	**-1,15**	**-1,29**	**-0,49**	**-2,07**	**-0,65**	**-0,15**	**-1,14**
**IL1beta**	**23,06**	**26,66**	**19,56**	**16,03**	**18,52**	**13,58**	-0,05	3,81	-3,76	**13,17**	**14,04**	**12,30**	**4,51**	**6,66**	**2,39**	**10,78**	**12,82**	**8,79**
**IL1RA**	**15,23**	**23,90**	**7,16**	**-7,25**	**-4,45**	**-9,97**	**9,36**	**15,59**	**3,47**	2,26	5,36	-0,74	**-7,96**	**-3,65**	**-12,07**	**-11,55**	**-8,20**	**-14,78**
**IL6**	**16,87**	**19,61**	**14,19**	**12,33**	**14,31**	**10,39**	1,53	4,91	-1,75	**8,60**	**9,38**	**7,82**	**3,27**	**4,82**	**1,74**	**7,29**	**8,78**	**5,83**
**IL8**	1,27	3,89	-1,28	-0,54	0,37	-1,44	**1,55**	**2,66**	**0,44**	0,07	0,57	-0,44	**-1,50**	**-0,76**	**-2,24**	0,01	0,46	-0,45
**Leptin**	**24,15**	**29,77**	**18,76**	**16,48**	**19,08**	**13,93**	-2,60	2,16	-7,15	**12,42**	**14,38**	**10,49**	**3,00**	**5,88**	**0,20**	**15,37**	**18,14**	**12,66**
**MCP1**	3,39	9,41	-2,30	**-2,75**	**-1,32**	**-4,16**	**5,11**	**7,24**	**3,01**	**-3,89**	**-3,38**	**-4,40**	-0,98	0,17	-2,12	**-3,59**	**-2,81**	**-4,36**
**TNFalfa**	2,42	6,03	-1,06	**-1,82**	**-0,96**	**-2,68**	**1,38**	**2,43**	**0,34**	-1,05	0,06	-2,14	**-1,51**	**-0,63**	**-2,39**	**-1,62**	**-1,08**	**-2,16**

Beta estimates and 95% Credible intervals expressed as percent change of child protein levels per quartile increase in the exposure mixture. Significant associations with an FDR p-value below 0.05 are bolded. All models were adjusted for subcohort, parental country of birth, maternal age, maternal education level, maternal pre-pregnancy body mass index, parity, maternal smoking in pregnancy, and maternal fish intake in pregnancy. Abbreviations: APO, apolipoprotein; BAFF, B-cell activating factor; BWQS, bayesian weighted quantile sum; CRP, C reactive protein; EDC: endocrine disrupting chemicals; HGF, hepatocyte growth factor; HMWPs, high-molecular weight phthalates; IL, interleukin; IL1RA, interleukin 1 receptor antagonist; LCrI, lower 95% credible interval; LMWPs, low-molecular-weight phthalate metabolites; MCP1, Monocyte Chemoattractant Protein-1; OCs, organochlorines; PBDEs, polybrominated diethyl ethers; TNF, Tumour Necrosis Factor; UCrI, upper 95% credible interval.

© 2024 Güil-Oumrait N et al. *JAMA Network Open.*

eTable 12. Adjusted Associations Between Prenatal EDC Mixtures and Child Serum Metabolites Using BWQS

	Metals mixture			OCs mixture			PBDEs mixture			PFASs mixture			LMWPs mixture			HMWPs mixture		
**Omics**	**β**	**LCrI**	**UCrI**	**β**	**LCrI**	**UCrI**	**β**	**LCrI**	**UCrI**	**β**	**LCrI**	**UCrI**	**β**	**LCrI**	**UCrI**	**β**	**LCrI**	**UCrI**
ADMA	**-1,61**	**-0,47**	**-2,73**	**1,17**	**1,59**	**0,76**	**1,62**	**2,26**	**0,98**	**-1,00**	**-0,75**	**-1,24**	0,07	0,47	-0,33	**-0,99**	**-0,42**	**-1,56**
Ala	-0,24	0,89	-1,36	**0,90**	**1,61**	**0,20**	0,22	0,92	-0,48	**2,03**	**2,26**	**1,81**	**0,64**	**1,12**	**0,16**	**0,89**	**1,48**	**0,31**
alpha.AAA	**8,19**	**12,22**	**4,31**	**5,92**	**7,22**	**4,64**	**3,97**	**6,26**	**1,73**	**5,24**	**6,55**	**3,95**	-0,59	0,77	-1,94	**1,79**	**3,10**	**0,50**
Arg	**2,15**	**3,61**	**0,71**	**1,88**	**2,47**	**1,30**	**2,01**	**2,79**	**1,24**	**2,98**	**3,28**	**2,68**	0,47	1,08	-0,13	-0,22	0,47	-0,91
Asp	**7,03**	**9,21**	**4,89**	**-4,29**	**-3,51**	**-5,06**	**4,98**	**7,04**	**2,97**	**-3,11**	**-2,27**	**-3,94**	**-3,17**	**-1,76**	**-4,57**	**-7,38**	**-6,59**	**-8,17**
C0	-0,72	0,38	-1,81	0,66	1,51	-0,18	-0,31	0,43	-1,04	**2,24**	**2,48**	**2,01**	0,57	1,08	0,05	**1,80**	**2,27**	**1,32**
C10	**-7,20**	**-5,81**	**-8,56**	0,04	1,74	-1,63	1,87	3,67	0,11	**-3,60**	**-3,30**	**-3,90**	0,59	1,68	-0,48	**-1,50**	**-0,65**	**-2,34**
C10.1	**-7,44**	**-5,84**	**-9,01**	**3,81**	**4,75**	**2,87**	**4,08**	**5,76**	**2,42**	**-5,38**	**-5,03**	**-5,73**	**1,45**	**2,38**	**0,53**	-0,22	0,94	-1,35
C12	**-7,64**	**-6,58**	**-8,70**	-1,76	0,09	-3,58	**1,70**	**3,16**	**0,25**	**-3,76**	**-3,48**	**-4,04**	0,18	1,18	-0,81	**-2,00**	**-1,10**	**-2,90**
C12.1	**-5,09**	**-3,68**	**-6,48**	0,16	1,19	-0,87	**2,07**	**3,25**	**0,91**	**-3,00**	**-2,73**	**-3,28**	-0,23	0,42	-0,87	**-1,24**	**-0,63**	**-1,84**
C14	**-3,83**	**-2,97**	**-4,69**	-1,02	0,02	-2,04	0,56	1,66	-0,53	**-1,66**	**-1,38**	**-1,93**	-0,49	0,22	-1,21	**-1,01**	**-0,41**	**-1,61**
C14.1	**-6,20**	**-1,52**	**-10,65**	1,83	4,96	-1,21	**4,25**	**6,29**	**2,25**	**-5,58**	**-5,20**	**-5,95**	-0,14	1,09	-1,36	**-3,42**	**-2,20**	**-4,62**
C14.1.OH	**-2,89**	**-1,20**	**-4,55**	**1,03**	**1,72**	**0,34**	**1,69**	**2,48**	**0,91**	**-1,58**	**-1,34**	**-1,82**	0,44	1,02	-0,13	-0,47	0,05	-0,99
C14.2	**-8,96**	**-6,61**	**-11,25**	**2,87**	**4,71**	**1,06**	**4,99**	**7,02**	**3,00**	**-6,72**	**-6,35**	**-7,10**	0,38	1,47	-0,69	**-1,70**	**-0,64**	**-2,75**
C16	-3,30	0,04	-6,54	**2,36**	**4,48**	**0,29**	**3,14**	**4,56**	**1,74**	**-2,67**	**-2,43**	**-2,91**	0,68	1,39	-0,03	**-3,16**	**-2,57**	**-3,75**
C16.1	-1,83	0,61	-4,21	0,60	1,85	-0,64	**1,32**	**2,31**	**0,33**	**-2,51**	**-2,33**	**-2,69**	0,15	0,79	-0,48	**-1,30**	**-0,66**	**-1,94**
C16.1.OH	-2,12	0,49	-4,67	0,50	1,41	-0,40	**1,23**	**2,11**	**0,36**	**-1,72**	**-1,53**	**-1,90**	0,10	0,61	-0,40	-0,17	0,32	-0,66
C16.2	1,30	3,29	-0,64	-0,70	0,10	-1,50	**1,43**	**2,04**	**0,82**	**-1,68**	**-1,36**	**-2,01**	**-1,42**	**-0,95**	**-1,88**	**-1,97**	**-1,43**	**-2,51**
C18	**-3,47**	**-2,15**	**-4,78**	**-1,51**	**-0,44**	**-2,58**	**1,54**	**2,40**	**0,70**	**-1,11**	**-0,73**	**-1,50**	-0,19	0,38	-0,75	**-2,51**	**-2,00**	**-3,02**
C18.1	1,26	4,05	-1,46	1,61	3,39	-0,14	**3,75**	**5,04**	**2,48**	**-2,96**	**-2,72**	**-3,21**	**-1,43**	**-0,54**	**-2,32**	**-3,56**	**-2,95**	**-4,17**
C18.2	-2,72	3,20	-8,30	**3,31**	**3,87**	**2,75**	**4,73**	**5,80**	**3,68**	**-4,64**	**-4,39**	**-4,88**	-0,01	0,89	-0,91	**-1,89**	**-1,20**	**-2,59**
C2	-1,75	4,49	-7,63	**3,38**	**4,97**	**1,80**	**3,21**	**4,68**	**1,77**	**-4,07**	**-3,74**	**-4,41**	**-2,62**	**-1,68**	**-3,55**	**-3,66**	**-2,58**	**-4,73**
C3	0,91	2,84	-0,99	**2,70**	**3,71**	**1,69**	1,37	3,14	-0,38	**3,58**	**4,52**	**2,66**	0,51	1,72	-0,68	**1,78**	**2,64**	**0,93**
C3.OH	0,94	2,24	-0,34	-0,25	0,24	-0,75	0,05	1,09	-0,98	-0,24	0,12	-0,60	0,76	1,65	-0,12	0,32	0,97	-0,33
C4	-0,52	1,44	-2,46	-0,37	1,00	-1,71	-0,38	1,40	-2,12	**2,53**	**3,33**	**1,75**	1,04	2,65	-0,55	**3,03**	**3,77**	**2,30**

© 2024 Güil-Oumrait N et al. *JAMA Network Open.*

C5	0,79	4,28	-2,59	**3,09**	**3,91**	**2,27**	**1,66**	**3,15**	**0,19**	**3,97**	**4,57**	**3,38**	0,41	1,30	-0,47	**2,50**	**3,19**	**1,81**
C5.DC..C6.OH.	**-2,88**	**-2,06**	**-3,69**	**-0,90**	**-0,26**	**-1,53**	0,22	0,91	-0,47	**-1,41**	**-1,12**	**-1,70**	-0,39	0,07	-0,84	-0,03	0,52	-0,57
C7.DC	**-3,36**	**-2,13**	**-4,57**	**-0,93**	**-0,42**	**-1,44**	0,45	1,32	-0,41	**-0,67**	**-0,41**	**-0,93**	-0,69	-0,10	-1,28	**-1,11**	**-0,58**	**-1,64**
C8	**-4,29**	**-3,18**	**-5,38**	-0,06	1,16	-1,27	0,95	2,34	-0,41	**-2,07**	**-1,84**	**-2,30**	0,76	1,57	-0,04	**-0,78**	**-0,12**	**-1,43**
Glu	**-9,95**	**-8,55**	**-11,32**	**3,83**	**5,14**	**2,54**	**6,69**	**8,59**	**4,83**	**-7,21**	**-6,83**	**-7,58**	-0,44	1,40	-2,24	**-3,95**	**-2,83**	**-5,07**
H1	0,46	1,39	-0,45	**1,00**	**1,31**	**0,69**	0,02	0,32	-0,28	**1,33**	**1,48**	**1,17**	**0,45**	**0,77**	**0,13**	**0,48**	**0,67**	**0,28**
His	0,17	1,03	-0,69	0,01	0,52	-0,50	**0,72**	**1,34**	**0,11**	**1,13**	**1,30**	**0,96**	0,27	0,70	-0,15	**-0,65**	**-0,27**	**-1,02**
Ile	**6,82**	**9,23**	**4,47**	**3,82**	**4,80**	**2,86**	**4,09**	**5,49**	**2,70**	**6,62**	**7,12**	**6,11**	-1,02	-0,09	-1,95	-0,83	0,34	-1,98
Kynurenine	-1,43	-0,04	-2,81	0,19	0,87	-0,50	0,84	1,79	-0,10	0,28	0,59	-0,02	**2,01**	**2,69**	**1,33**	**1,05**	**1,53**	**0,58**
Leu	**5,78**	**7,60**	**3,99**	**3,18**	**4,24**	**2,13**	**3,00**	**4,32**	**1,70**	**5,64**	**6,11**	**5,17**	-0,37	0,59	-1,31	-0,25	0,90	-1,38
Lys	1,86	4,24	-0,47	**3,96**	**4,76**	**3,17**	**1,11**	**2,01**	**0,22**	**4,68**	**5,08**	**4,29**	**1,66**	**2,43**	**0,89**	**2,88**	**3,62**	**2,15**
lysoPC.a.C14.0	-0,82	-0,02	-1,60	-0,38	0,34	-1,10	**-0,98**	**-0,50**	**-1,46**	**0,93**	**1,10**	**0,75**	0,09	0,36	-0,18	-0,04	0,35	-0,43
lysoPC.a.C16.0	-0,51	0,72	-1,73	**-1,99**	**-1,27**	**-2,71**	**1,26**	**2,38**	**0,14**	**1,46**	**1,96**	**0,95**	0,13	0,96	-0,70	**-1,78**	**-1,20**	**-2,35**
lysoPC.a.C16.1	-1,89	0,38	-4,10	**-3,02**	**-2,41**	**-3,62**	**-1,90**	**-0,65**	**-3,13**	**3,00**	**3,39**	**2,62**	0,07	0,66	-0,52	**-1,07**	**-0,34**	**-1,80**
lysoPC.a.C17.0	-3,21	-0,45	-5,89	**-4,00**	**-3,13**	**-4,87**	**-3,29**	**-1,69**	**-4,86**	**1,61**	**1,97**	**1,24**	-0,46	0,31	-1,22	**-0,87**	**-0,15**	**-1,59**
lysoPC.a.C18.0	-0,81	0,71	-2,32	**-1,51**	**-0,56**	**-2,45**	**1,14**	**2,09**	**0,20**	0,07	0,51	-0,37	0,31	1,71	-1,07	**-1,63**	**-0,99**	**-2,27**
lysoPC.a.C20.3	**8,33**	**11,32**	**5,41**	0,57	1,94	-0,79	**5,65**	**8,21**	**3,16**	**6,90**	**7,43**	**6,38**	-1,65	0,64	-3,87	**-4,26**	**-3,08**	**-5,43**
lysoPC.a.C20.4	**4,32**	**7,23**	**1,48**	**1,99**	**2,84**	**1,15**	**4,82**	**6,25**	**3,41**	**3,83**	**4,43**	**3,24**	-1,24	0,46	-2,91	**-2,48**	**-1,79**	**-3,17**
lysoPC.a.C26.0	-0,03	2,58	-2,58	-0,78	0,29	-1,83	1,42	3,83	-0,94	0,46	1,61	-0,67	**-1,90**	**-0,83**	**-2,97**	**-2,29**	**-1,47**	**-3,10**
lysoPC.a.C28.0	-0,64	1,31	-2,56	-0,65	0,04	-1,35	0,85	2,56	-0,84	0,47	1,11	-0,16	**-1,19**	**-0,52**	**-1,86**	**-1,95**	**-1,44**	**-2,46**
lysoPC.a.C28.1	-0,55	1,74	-2,80	**-0,82**	**-0,09**	**-1,55**	-1,17	0,35	-2,67	**1,22**	**1,73**	**0,72**	**-0,71**	**-0,21**	**-1,21**	**-1,15**	**-0,59**	**-1,70**
Met	0,79	3,24	-1,60	**2,68**	**3,48**	**1,89**	**2,01**	**3,14**	**0,89**	**4,38**	**4,79**	**3,97**	**1,32**	**2,08**	**0,57**	**1,08**	**2,03**	**0,14**
Met.SO	**-8,44**	**-7,23**	**-9,63**	0,86	2,11	-0,38	**8,11**	**10,28**	**5,99**	**-6,02**	**-5,63**	**-6,41**	-0,86	0,37	-2,07	**-3,02**	**-1,84**	**-4,19**
Orn	0,73	2,59	-1,11	0,27	1,51	-0,96	**3,28**	**4,72**	**1,85**	**2,66**	**3,14**	**2,18**	-0,61	0,07	-1,28	**-1,31**	**-0,45**	**-2,16**
PC.aa.C24.0	-2,35	0,80	-5,41	-0,54	0,29	-1,37	0,63	2,48	-1,18	**-1,87**	**-0,98**	**-2,75**	**-1,90**	**-1,21**	**-2,59**	**-1,62**	**-0,90**	**-2,34**
PC.aa.C30.0	-0,73	1,08	-2,50	-0,93	0,31	-2,15	**-2,17**	**-0,80**	**-3,52**	**0,96**	**1,46**	**0,46**	0,70	1,85	-0,43	0,02	1,04	-1,00
PC.aa.C32.0	1,33	2,72	-0,05	-0,50	0,54	-1,54	**1,22**	**2,16**	**0,28**	-0,09	0,39	-0,58	-0,69	-0,13	-1,25	**-1,48**	**-1,03**	**-1,92**
PC.aa.C32.1	-2,59	0,58	-5,65	**-3,49**	**-2,38**	**-4,59**	**-4,29**	**-2,51**	**-6,04**	**2,62**	**3,10**	**2,14**	-0,10	0,99	-1,18	**-1,21**	**-0,44**	**-1,98**

© 2024 Güil-Oumrait N et al. *JAMA Network Open.*

PC.aa.C32.2	-0,73	1,35	-2,77	0,20	1,63	-1,21	**-1,90**	**-0,69**	**-3,10**	**0,88**	**1,44**	**0,32**	0,48	1,81	-0,84	-0,52	0,30	-1,33
PC.aa.C32.3	-0,88	1,27	-2,98	**-1,99**	**-1,21**	**-2,76**	**-3,03**	**-2,17**	**-3,88**	**1,19**	**1,60**	**0,78**	-0,18	0,71	-1,05	-0,43	0,08	-0,93
PC.aa.C34.3	**-5,14**	**-3,29**	**-6,95**	**-5,78**	**-4,96**	**-6,59**	**-3,29**	**-1,74**	**-4,82**	-0,75	0,02	-1,51	**-1,24**	**-0,32**	**-2,15**	**-2,96**	**-2,29**	**-3,62**
PC.aa.C34.4	1,83	4,19	-0,48	0,19	1,57	-1,17	**-2,33**	**-1,17**	**-3,48**	**2,96**	**3,45**	**2,47**	0,00	1,17	-1,14	0,12	1,09	-0,84
PC.aa.C36.0	-0,27	3,40	-3,81	**-2,16**	**-1,16**	**-3,16**	**-3,27**	**-2,10**	**-4,42**	**2,66**	**3,24**	**2,09**	0,96	2,00	-0,07	**1,45**	**2,21**	**0,70**
PC.aa.C36.1	2,22	4,40	0,08	**-1,99**	**-0,97**	**-3,01**	-0,71	0,54	-1,94	**2,37**	**2,66**	**2,07**	-0,68	0,00	-1,35	**-1,74**	**-1,08**	**-2,39**
PC.aa.C36.3	**3,02**	**4,80**	**1,27**	-0,02	1,20	-1,22	**2,46**	**3,51**	**1,42**	**1,40**	**1,90**	**0,90**	**-0,83**	**-0,22**	**-1,44**	**-1,99**	**-1,35**	**-2,63**
PC.aa.C36.5	-8,30	6,78	-21,25	**-15,10**	**-13,94**	**-16,25**	**-12,42**	**-9,49**	**-15,25**	**8,26**	**8,76**	**7,77**	**-3,37**	**-1,70**	**-5,02**	**-3,42**	**-1,90**	**-4,91**
PC.aa.C36.6	**8,64**	**13,31**	**4,17**	**-7,69**	**-6,66**	**-8,71**	**-10,49**	**-8,69**	**-12,26**	**8,41**	**8,78**	**8,05**	-0,22	1,54	-1,95	0,59	1,98	-0,78
PC.aa.C38.0	-0,40	5,08	-5,59	**-2,45**	**-1,48**	**-3,41**	**-3,34**	**-1,83**	**-4,81**	**2,98**	**3,60**	**2,37**	0,85	2,04	-0,33	**2,91**	**3,71**	**2,13**
PC.aa.C38.1	-1,46	5,75	-8,17	**-6,74**	**-5,88**	**-7,60**	**-4,61**	**-2,95**	**-6,25**	0,15	2,66	-2,29	-0,49	0,72	-1,67	**2,63**	**3,62**	**1,65**
PC.aa.C38.3	**5,83**	**7,31**	**4,36**	0,75	1,71	-0,21	**1,32**	**2,55**	**0,11**	**2,35**	**2,72**	**1,97**	0,62	1,80	-0,55	-0,39	0,45	-1,21
PC.aa.C38.4	**4,25**	**5,94**	**2,59**	**2,22**	**2,98**	**1,48**	**2,16**	**3,06**	**1,26**	**0,54**	**1,02**	**0,07**	0,57	1,48	-0,34	0,26	0,88	-0,35
PC.aa.C40.1	-1,46	0,08	-2,97	**-2,00**	**-1,60**	**-2,39**	**-1,13**	**-0,46**	**-1,78**	**-1,76**	**-1,50**	**-2,02**	0,15	0,64	-0,35	-0,05	0,27	-0,37
PC.aa.C40.4	2,08	4,36	-0,15	**1,80**	**2,52**	**1,09**	**2,14**	**3,14**	**1,16**	**-0,43**	**-0,12**	**-0,75**	0,67	1,42	-0,07	-0,02	0,46	-0,50
PC.aa.C40.5	**-3,32**	**-1,00**	**-5,59**	**-2,92**	**-2,20**	**-3,63**	**-2,04**	**-0,74**	**-3,33**	**-2,00**	**-1,59**	**-2,40**	-0,33	0,33	-0,98	**-1,45**	**-0,98**	**-1,91**
PC.aa.C40.6	4,25	14,58	-5,15	**-4,47**	**-3,67**	**-5,26**	**-7,28**	**-5,96**	**-8,58**	**5,39**	**5,77**	**5,01**	1,42	2,66	0,20	**1,99**	**3,16**	**0,83**
PC.aa.C42.0	-1,66	1,15	-4,40	**-2,72**	**-2,20**	**-3,23**	**-2,34**	**-1,35**	**-3,33**	**-1,87**	**-1,33**	**-2,41**	-0,01	0,59	-0,61	**1,24**	**1,98**	**0,50**
PC.aa.C42.1	-1,65	0,48	-3,73	**-1,93**	**-1,33**	**-2,52**	**-1,60**	**-0,65**	**-2,54**	**-0,99**	**-0,48**	**-1,49**	0,66	1,30	0,01	**0,84**	**1,36**	**0,33**
PC.ae.C30.2	-0,22	1,98	-2,37	0,41	1,01	-0,18	**-1,69**	**-0,66**	**-2,72**	**1,02**	**1,47**	**0,57**	**0,83**	**1,42**	**0,24**	**1,10**	**1,60**	**0,60**
PC.ae.C32.1	0,80	1,89	-0,27	0,29	0,87	-0,29	0,38	1,14	-0,37	**0,90**	**1,49**	**0,31**	0,41	1,20	-0,38	0,13	0,62	-0,35
PC.ae.C32.2	**2,68**	**4,32**	**1,07**	-0,62	0,19	-1,43	**-1,51**	**-0,77**	**-2,24**	**2,29**	**2,78**	**1,80**	0,07	0,86	-0,72	**0,64**	**1,15**	**0,13**
PC.ae.C34.0	0,90	3,84	-1,96	**-2,36**	**-1,33**	**-3,37**	**-3,31**	**-1,87**	**-4,74**	**2,75**	**3,23**	**2,29**	-0,27	1,41	-1,93	-0,21	0,46	-0,88
PC.ae.C34.1	0,44	1,90	-1,01	**-1,15**	**-0,48**	**-1,81**	-0,35	0,73	-1,42	0,37	0,73	0,01	-0,75	0,02	-1,51	**-0,83**	**-0,40**	**-1,25**
PC.ae.C34.2	1,70	3,48	-0,05	**1,96**	**2,62**	**1,29**	**2,08**	**3,06**	**1,11**	**1,40**	**2,53**	**0,29**	0,50	1,55	-0,54	**1,73**	**2,43**	**1,04**
PC.ae.C34.3	1,14	2,56	-0,26	0,57	1,47	-0,31	**1,56**	**2,36**	**0,76**	**1,38**	**2,40**	**0,37**	0,85	1,67	0,05	0,08	0,70	-0,53
PC.ae.C36.0	0,14	3,07	-2,71	**-2,56**	**-1,81**	**-3,30**	**-1,73**	**-0,78**	**-2,66**	**1,25**	**1,68**	**0,83**	-0,78	0,17	-1,72	-0,30	0,30	-0,91
PC.ae.C36.2	-1,25	0,08	-2,55	**-1,67**	**-0,89**	**-2,43**	-1,04	-0,04	-2,04	-0,28	0,04	-0,60	-0,39	0,55	-1,32	-0,55	-0,03	-1,06

© 2024 Güil-Oumrait N et al. *JAMA Network Open.*

PC.ae.C36.3	1,77	3,58	0,00	**1,06**	**1,73**	**0,39**	**1,83**	**2,75**	**0,93**	**1,08**	**1,86**	**0,31**	0,31	1,24	-0,61	**1,11**	**1,74**	**0,49**
PC.ae.C36.4	**5,56**	**7,32**	**3,83**	**4,67**	**5,40**	**3,93**	**3,65**	**5,22**	**2,12**	**3,62**	**4,58**	**2,66**	0,71	1,71	-0,27	**2,77**	**3,54**	**2,00**
PC.ae.C36.5	**4,49**	**6,32**	**2,69**	**2,45**	**3,23**	**1,68**	1,06	2,12	0,02	**3,30**	**4,11**	**2,50**	**1,50**	**2,25**	**0,76**	**2,26**	**3,15**	**1,38**
PC.ae.C38.4	0,55	2,00	-0,89	**1,56**	**2,04**	**1,08**	**1,70**	**2,63**	**0,77**	0,32	0,96	-0,31	0,39	1,23	-0,44	**0,67**	**1,20**	**0,15**
PC.ae.C38.5	0,88	3,22	-1,40	**2,20**	**2,65**	**1,74**	**2,20**	**3,29**	**1,13**	-0,04	0,59	-0,66	0,22	0,99	-0,55	**1,64**	**2,20**	**1,08**
PC.ae.C38.6	1,20	4,22	-1,73	-1,02	0,27	-2,30	**-2,63**	**-1,24**	**-4,00**	**3,54**	**4,19**	**2,90**	1,11	2,31	-0,07	**3,01**	**3,93**	**2,10**
PC.ae.C40.4	-0,04	1,74	-1,79	**0,81**	**1,24**	**0,39**	**2,10**	**3,15**	**1,05**	**-1,39**	**-1,13**	**-1,64**	0,49	1,34	-0,35	0,26	0,64	-0,11
PC.ae.C40.5	-1,30	0,40	-2,97	**-0,94**	**-0,59**	**-1,30**	0,13	1,27	-1,00	**-1,45**	**-1,19**	**-1,70**	0,03	0,71	-0,64	0,35	0,72	-0,02
PC.ae.C40.6	-3,69	0,51	-7,71	**-3,62**	**-2,95**	**-4,29**	**-4,63**	**-3,31**	**-5,93**	0,77	3,16	-1,56	0,54	1,93	-0,84	**1,84**	**2,65**	**1,03**
PC.ae.C42.3	-1,71	1,06	-4,40	**-3,15**	**-2,62**	**-3,68**	**-1,19**	**-0,27**	**-2,10**	**-1,46**	**-0,88**	**-2,03**	-0,01	0,56	-0,58	-0,32	0,23	-0,86
PC.ae.C42.4	**-2,01**	**-0,45**	**-3,54**	**-0,42**	**-0,04**	**-0,80**	1,19	2,50	-0,10	**-0,83**	**-0,53**	**-1,14**	0,49	1,30	-0,31	**0,52**	**0,99**	**0,06**
PC.ae.C42.5	-0,71	1,11	-2,50	**-0,69**	**-0,24**	**-1,14**	0,45	1,55	-0,64	**-1,11**	**-0,81**	**-1,41**	-0,04	0,56	-0,64	**1,22**	**1,66**	**0,79**
PC.ae.C44.4	-0,49	0,93	-1,89	**-1,09**	**-0,56**	**-1,62**	0,18	1,17	-0,80	0,06	0,35	-0,23	-0,28	0,20	-0,75	0,12	0,53	-0,28
PC.ae.C44.5	-0,20	2,06	-2,41	-0,55	0,20	-1,29	0,04	1,46	-1,37	-0,07	0,38	-0,52	-0,23	0,54	-0,99	**1,46**	**2,07**	**0,86**
PC.ae.C44.6	-2,18	-0,33	-4,00	0,16	0,99	-0,67	0,06	2,04	-1,87	**-1,94**	**-1,54**	**-2,34**	0,72	1,48	-0,03	**2,17**	**2,83**	**1,51**
Phe	**3,45**	**5,49**	**1,45**	**1,28**	**2,16**	**0,41**	**2,98**	**3,95**	**2,02**	**3,39**	**3,72**	**3,07**	**-0,85**	**-0,21**	**-1,48**	**-1,49**	**-0,82**	**-2,16**
Pro	-0,50	1,35	-2,32	1,18	2,36	0,02	1,02	2,17	-0,13	**4,08**	**4,55**	**3,62**	-0,13	0,68	-0,92	0,30	1,13	-0,52
SDMA	-4,31	1,56	-9,84	**-3,08**	**-0,84**	**-5,27**	1,15	4,49	-2,08	**-3,90**	**-1,91**	**-5,85**	0,86	2,96	-1,20	**-6,59**	**-4,68**	**-8,45**
SM..OH..C16.1	-2,15	0,84	-5,06	0,17	1,15	-0,80	**-3,08**	**-1,99**	**-4,16**	**2,56**	**3,08**	**2,05**	**2,05**	**3,05**	**1,06**	**3,22**	**4,09**	**2,36**
SM..OH..C24.1	1,91	4,31	-0,44	**1,45**	**2,16**	**0,73**	0,55	1,59	-0,49	0,36	0,79	-0,07	**1,87**	**2,52**	**1,23**	**1,52**	**2,35**	**0,69**
SM.C16.0	-0,09	0,85	-1,03	**0,92**	**1,27**	**0,57**	**1,22**	**1,72**	**0,71**	-0,14	0,11	-0,40	**1,39**	**1,84**	**0,94**	**0,90**	**1,33**	**0,47**
SM.C20.2	-1,16	0,64	-2,93	**2,59**	**3,22**	**1,97**	**1,58**	**2,63**	**0,55**	**-0,89**	**-0,48**	**-1,31**	**1,58**	**2,45**	**0,71**	**1,99**	**2,65**	**1,34**
SM.C24.1	-1,91	-0,21	-3,58	**-1,02**	**-0,44**	**-1,60**	-0,58	0,47	-1,61	**-0,79**	**-0,37**	**-1,21**	**1,92**	**2,62**	**1,22**	**1,44**	**2,06**	**0,82**
SM.C26.0	-1,48	1,16	-4,06	-1,12	-0,10	-2,12	**-1,40**	**-0,22**	**-2,58**	-0,46	0,30	-1,21	0,72	1,65	-0,21	**1,34**	**2,04**	**0,65**
SM.C26.1	**-4,28**	**-2,16**	**-6,36**	**-2,66**	**-1,61**	**-3,70**	**-2,57**	**-1,66**	**-3,46**	**-2,16**	**-1,26**	**-3,06**	**2,92**	**3,70**	**2,15**	**2,93**	**3,93**	**1,93**
Thr	**4,41**	**5,95**	**2,90**	**3,18**	**3,80**	**2,56**	**1,08**	**1,97**	**0,20**	**3,74**	**4,03**	**3,44**	**1,22**	**1,91**	**0,53**	**1,55**	**2,26**	**0,83**
Trp	2,04	4,24	-0,10	**0,95**	**1,59**	**0,31**	**1,73**	**2,61**	**0,86**	**2,97**	**3,24**	**2,71**	0,62	1,22	0,03	-0,17	0,45	-0,77
Tyr	**3,23**	**5,59**	**0,93**	**2,00**	**2,99**	**1,02**	**2,80**	**4,12**	**1,50**	**3,35**	**3,73**	**2,97**	**0,78**	**1,39**	**0,17**	0,25	1,19	-0,68

© 2024 Güil-Oumrait N et al. *JAMA Network Open.*

Val	**4,99**	**6,37**	**3,63**	**2,62**	**3,44**	**1,81**	**2,40**	**3,38**	**1,44**	**4,59**	**4,96**	**4,21**	-0,64	0,06	-1,34	0,03	0,78	-0,71

Beta estimates and 95% Credible intervals expressed as percent change of child protein levels per quartile increase in the exposure mixture. Significant associations with an FDR p-value below 0.05 are bolded. All models were adjusted for subcohort, parental country of birth, maternal age, maternal education level, maternal pre-pregnancy body mass index, parity, maternal smoking in pregnancy, and maternal fish intake in pregnancy. Abbreviations: BWQS, bayesian weighted quantile sum; EDC: endocrine disrupting chemicals; HMWPs, high-molecular weight phthalates; LCrI, lower 95% credible interval; LMWPs, low-molecular-weight phthalate metabolites; OCs, organochlorines; PBDEs, polybrominated diethyl ethers; UCrI, upper 95% credible interval.

© 2024 Güil-Oumrait N et al. *JAMA Network Open.*

eTable 13. Adjusted Associations Between Prenatal EDC Mixtures and Child Urine Metabolites Using BWQS

	**Metals mixture**			**OCs mixture**			**PBDEs mixture**			**PFASs mixture**			**LMWPs mixture**			**HMWPs mixture**		
**Omics**	**β**	**LCrI**	**UCrI**	**β**	**LCrI**	**UCrI**	**β**	**LCrI**	**UCrI**	**β**	**LCrI**	**UCrI**	**β**	**LCrI**	**UCrI**	**β**	**LCrI**	**UCrI**
**Acetone**	-3,83	2,74	-9,98	-0,49	2,17	-3,08	**6,39**	**11,22**	**1,78**	**-8,55**	**-7,48**	**-9,60**	-2,14	0,78	-4,97	1,24	3,25	-0,73
**Alanine**	-1,55	1,53	-4,54	-1,12	0,09	-2,32	-1,38	0,29	-3,02	0,05	0,71	-0,61	-0,11	1,03	-1,24	-0,04	0,95	-1,02
**Hippurate**	**-25,65**	**-22,76**	**-28,44**	**-10,09**	**-7,69**	**-12,43**	**-11,25**	**-7,62**	**-14,74**	**-4,67**	**-2,31**	**-6,98**	1,30	4,00	-1,34	**-4,85**	**-2,22**	**-7,41**
**Leucine**	1,15	2,63	-0,31	**3,21**	**4,18**	**2,24**	0,45	1,76	-0,84	**2,41**	**2,91**	**1,90**	-0,61	0,19	-1,40	**1,17**	**1,79**	**0,55**
**p.cresol.sulfate**	-2,76	3,47	-8,62	**-2,70**	**-0,53**	**-4,83**	2,64	6,46	-1,04	-1,56	-0,05	-3,04	-1,37	1,56	-4,22	0,52	2,88	-1,79
**Tyrosine**	0,75	4,04	-2,44	1,25	2,54	-0,03	0,46	1,85	-0,90	0,18	0,79	-0,42	-0,48	0,33	-1,28	0,74	1,94	-0,45
**Valine**	0,32	1,60	-0,94	**0,73**	**1,41**	**0,06**	0,58	1,46	-0,31	**-1,38**	**-1,09**	**-1,67**	**-1,20**	**-0,43**	**-1,96**	**-0,87**	**-0,50**	**-1,25**
**3-Aminoisobutyrate**	**-10,71**	**-4,09**	**-16,87**	**4,49**	**8,75**	**0,40**	-2,33	1,95	-6,42	**-1,96**	**-0,48**	**-3,42**	**5,18**	**9,06**	**1,44**	**-3,06**	**-0,48**	**-5,58**
**3-Hydroxyisobutyrate**	**5,84**	**8,33**	**3,41**	**2,51**	**3,98**	**1,05**	-0,05	1,31	-1,40	**4,91**	**5,41**	**4,42**	**1,63**	**2,93**	**0,34**	**3,47**	**4,65**	**2,30**
**4-Deoxyerythronic.acid**	**7,87**	**9,85**	**5,93**	**5,98**	**7,42**	**4,56**	1,32	3,21	-0,53	**6,76**	**7,37**	**6,16**	1,60	3,12	0,11	1,18	2,51	-0,13

Beta estimates and 95% Credible intervals expressed as percent change of child urine metabolite levels per quartile increase in the exposure mixture. Significant associations with an FDR p-value below 0.05 are bolded. All models were adjusted for subcohort, parental country of birth, maternal age, maternal education level, maternal pre-pregnancy body mass index, parity, maternal smoking in pregnancy, and maternal fish intake in pregnancy. Abbreviations: BWQS, bayesian weighted quantile sum; EDC: endocrine disrupting chemicals; HMWPs, high-molecular weight phthalates; LCrI, lower 95% credible interval; LMWPs, low-molecular-weight phthalate metabolites; OCs, organochlorines; PBDEs, polybrominated diethyl ethers; UCrI, upper 95% credible interval.

© 2024 Güil-Oumrait N et al. *JAMA Network Open.*

eTable 14. Environmental-Wide Association Study (ExWAS) of Prenatal Individual EDCs and Child MetS

Exposure group	Exposure variable	MetS score		
		All (n=1134)		
		β (95% CI)	p-value	FDR-adjusted p-value
Metals	As	0.01 (-0.13, 0.15)	0.91	0.95
	Cd	0.12 (-0.13, 0.37)	0.35	0.95
	Co	0.00 (-0.31, 0.31)	0.99	0.95
	Cs	0.33 (-0.21, 0.86)	0.23	0.95
	Cu	-0.56 (-1.71, 0.59)	0.34	0.95
	Hg	0.16 (-0.09, 0.42)	0.21	0.95
	Mn	0.11 (-0.47, 0.69)	0.70	0.98
	Mo	0.10 (-0.22, 0.41)	0.55	0.95
	Pb	-0.01 (-0.38, 0.37)	0.97	0.99
OC pesticides	DDE	-0.06 (-0.22, 0.11)	0.49	0.95
	DDT	0.05 (-0.10, 0.20)	0.54	0.95
	HCB	0.06 (-0.21, 0.33)	0.67	0.98
PCBs	PCB118	-0.07 (-0.39, 0.26)	0.69	0.98
	PCB138	0.09 (-0.20, 0.38)	0.54	0.95
	PCB153	0.14 (-0.17, 0.45)	0.37	0.95
	PCB170	0.06 (-0.17, 0.29)	0.58	0.95
	PCB180	0.10 (-0.16, 0.36)	0.44	0.95
PBDEs	PBDE47	-0.01 (-0.20, 0.18)	0.92	0.99
	PBDE153	-0.02 (-0.11, 0.06)	0.59	0.95
PFASs	PFHxS	0.03 (-0.18, 0.24)	0.78	0.98
	PFNA	0.12 (-0.15, 0.39)	0.38	0.95
	PFOA	0.10 (-0.18, 0.38)	0.50	0.95
	PFOS	0.08 (-0.21, 0.38)	0.57	0.95
	PFUNDA	-0.02 (-0.30, 0.25)	0.88	0.99
LMWPs	MEP	-0.03 (-0.13, 0.08)	0.64	0.98
	MiBP	-0.08 (-0.27, 0.11)	0.41	0.95
	MnBP	-0.17 (-0.34, -0.01)	0.03	0.95
HMWPs	MBzP	-0.03 (-0.15, 0.09)	0.59	0.95
	MEHP	-0.13 (-0.28, 0.02)	0.09	0.95
	MEHHP	-0.13 (-0.29, 0.03)	0.10	0.95
	MEOHP	-0.14 (-0.30, 0.02)	0.08	0.95
	MECPP	-0.08 (-0.26, 0.10)	0.36	0.95
	oh-MiNP	-0.13 (-0.34, 0.08)	0.22	0.95
	oxo-MiNP	-0.05 (-0.21, 0.11)	0.51	0.95
Phenols	BPA	0.00 (-0.14, 0.14)	0.97	0.99
	OXBE	-0.02 (-0.07, 0.04)	0.58	0.95
	TRCS	0.01 (-0.04, 0.06)	0.75	0.98
Parabens	MEPA	0.03 (-0.05, 0.11)	0.44	0.95
	ETPA	0.01 (-0.06, 0.07)	0.84	0.99
	PRPA	0.03 (-0.05, 0.10)	0.47	0.95

© 2024 Güil-Oumrait N et al. *JAMA Network Open.*

	BUPA	-0.01 (-0.07, 0.06)	0.89	0.99
OP Pesticide metabolites				0.98
	DEP	0.02 (-0.13, 0.18)	0.78	
	DETP	0.00 (-0.07, 0.06)	0.91	0.99
	DMP	-0.02 (-0.17, 0.13)	0.79	0.98
	DMTP	0.04 (-0.07, 0.14)	0.50	0.95

Effect estimates were expressed per doubling of prenatal chemical levels. All models were adjusted for subcohort, parental country of birth, maternal age, maternal education level, maternal pre-pregnancy body mass index, parity, maternal smoking in pregnancy, and maternal fish intake in pregnancy. Abbreviations: As, inorganic arsenic; BPA, bisphenol A; BUPA, N-butyl paraben; Cd, cadmium; CI, confidence interval; Co, cobalt; Cs, caesium; Cu, copper; DDE, dichlorodiphenyldichloroethylene; DDT, dichlorodiphenyltrichloroethane; DEP, diethyl phosphate; DETP, diethylthiophosphate; DMP, dimethyl phosphate; DMTP, dimethylthiophosphate; EDC, endocrine-disrupting chemical; ETPA, ethyl paraben; FDR, false discovery rate; HCB, hexachlorobenzene; Hg, mercury; HMWPs, high-molecular-weight phthalate metabolites; LMWPs, low-molecular-weight phthalate metabolites; MBzP, monobenzylphthalate; MECPP,mono(2-ethyl-5-carboxypentyl) phthalate; MEHHP, mono(2-ethyl-5-hydroxyhexyl) phthalate; MEHP, mono-2-ethylhexyl phthalate; MEOHP, mono(2-ethyl-5-oxohexyl) phthalate; MEP, monoethyl phthalate; MEPA, methyl paraben; MetS, Metabolic Syndrome; MiBP, mono-iso-butyl phthalate; Mn, manganese; MnBP, mono-n-butyl phthalate; Mo, molybdenum; OC, organochlorine; OHMiNP, mono-hydroxy-isononyl phthalate; OP, organophosphate; OXBE, oxybenzone; OXOMiNP, mono-oxo-isononyl phthalate; Pb, lead; PBDEs, polybrominated diphenyl ethers; PCBs, polychlorinated biphenyls; PFASs, perfluoroalkyl substances; PFHxS, perfluorohexane sulfonate; PFNA, perfluorononanoic acid; PFOA, perfluoro-octanoic acid; PFOS, perfluoro-octane sulfonate; PFUnDA, perfluoroundecanoic acid; PRPA, propyl paraben; and TCS, triclosan

© 2024 Güil-Oumrait N et al. *JAMA Network Open.*

eTable 15. Sensitivity Analysis. BWQS Models of Lipophilic Mixture Groups and MetS Stratified by Gestational Weight Gain Category

Exposure		All (n=1134)	Gestational weight gain status: Excessive (n= 631)	Low/Adequate (n= 503)
OC pesticides	β (95% CrI)	0.22 (0.15, 0.29)	0.08 (-0.06,0.22)	0.32 (0.23, 0.40)
DDE	Weight (95% CrI)	0.27 (0.15, 0.38)	0.31 (0.24, 0.39)	0.27 (0.14, 0.40)
DDT		0.22 (0.08, 0.36)	0.33 (0.25, 0.41)	0.22 (0.12, 0.31)
HCB		0.51 (0.37, 0.66)	0.36 (0.29, 0.43)	0.51 (0.37,0.66)
PBDEs	β (95% CrI)	0.17 (0.06, 0.27)	0.21 (0.06, 0.36)	0.14 (0.02, 0.27)
PBDE47	Weight (95% CrI)	0.53 (0.24, 0.82)	0.53 (0.35, 0.72)	0.50 (0.27, 0.73)
PBDE153		0.47 (0.18, 0.76)	0.47 (0.28, 0.65)	0.50 (0.27, 0.73)

All models were adjusted for subcohort, parental country of birth, maternal age, maternal education level, maternal pre-pregnancy body mass index, parity, maternal smoking in pregnancy, and maternal fish intake in pregnancy. Abbreviations: BWQS, Bayesian weighted quantile sum; CrI, credible interval; DDE, dichlorodiphenyldichloroethylene; DDT, dichlorodiphenyltrichloroethane; HCB, hexachlorobenzene; MetS, Metabolic Syndrome; OC, organochlorine; PBDEs, polybrominated diphenyl ethers.

© 2024 Güil-Oumrait N et al. *JAMA Network Open.*

eTable 16. Sensitivity Analyses. BWQS Models of Phthalate and Non-Persistent Chemical Mixtures

		MetS score (n=1134)
**Phthalate metabolites mixture**	β (95% CrI)	-0.12 (-0.18, -0.07)
MEP	Weight (95% CrI)	0.14 (0.12, 0.16)
MiBP		0.17 (0.14, 0.20)
MnBP		0.23 (0.18, 0.29)
MBzP		0.13 (0.12, 0.15)
∑DEHP		0.19 (0.16, 0.23)
∑DiNP		0.13 (0.10, 0.15)
Nonpersistent mixture	β (95% CrI)	-0.15 (-0.23, -0.06)
MEP	Weight (95% CrI)	0.07 (0.06, 0.08)
MiBP		0.07 (0.06, 0.09)
MnBP		0.09 (0.07, 0.11)
MBzP		0.07 (0.06, 0.07)
∑DEHP		0.08 (0.07, 0.09)
∑DiNP		0.06 (0.05, 0.07)
BPA		0.08 (0.06, 0.10)
OXBE		0.08 (0.07, 0.10)
TRCS		0.06 (0.05, 0.07)
∑Parabens		0.07 (0.06, 0.09)
DEP		0.07 (0.06, 0.07)
DETP		0.07 (0.06, 0.08)
DMP		0.06 (0.05, 0.07)
DMTP		0.06 (0.05, 0.07)

All models were adjusted for subcohort, parental country of birth, maternal age, maternal education level, maternal pre-pregnancy body mass index, parity, maternal smoking in pregnancy, and maternal fish intake in pregnancy. Abbreviations: BPA, bisphenol A; CrI, credible interval; DEHP, Di(2-ethylhexyl) phthalate; DEP, diethyl phosphate; DETP, diethylthiophosphate; DiNP, Diisononyl phthalate; DMP, dimethyl phosphate; DMTP, dimethylthiophosphate; MBzP, monobenzylphthalate; MEP, monoethyl phthalate; MetS, Metabolic Syndrome; MiBP, mono-iso-butyl phthalate; MnBP, mono-n-butyl phthalate; OXBE, oxybenzone; TCS, triclosan.

© 2024 Güil-Oumrait N et al. *JAMA Network Open.*

eTable 17. Sensitivity Analyses. BWQS Models of Metals and Persistent Chemicals Mixture

Metals and persistent chemicals		MetS score (n=1134)
	β (95% CrI)	0.63 (0.47, 0.78)
As	Weight (95% CrI)	0.05 (0.02, 0.7)
Cd		0.07 (0.04, 0.11)
Co		0.05 (0.02, 0.07)
Cs		0.03 (0.02, 0.04)
Cu		0.03 (0.02, 0.05)
Hg		0.12 (0.06, 0.18)
Mn		0.03 (0.02, 0.05)
Mo		0.05 (0.03, 0.07)
Pb		0.04 (0.03, 0.05)
DDE		0.05 (0.03, 0.06)
DDT		0.04 (0.02, 0.07)
HCB		0.07 (0.04, 0.10)
∑PCBs		0.03 (0.02, 0.03)
PBDE47		0.06 (0.02, 0.11)
PBDE153		0.06 (0.02, 0.11)
PFHxS		0.03 (0.03, 0.04)
PFNA		0.08 (0.06, 0.10)
PFOA		0.04 (0.03, 0.05)
PFOS		0.03 (0.02, 0.03)
PFUNDA		0.03 (0.02, 0.04)

All models were adjusted for subcohort, parental country of birth, maternal age, maternal education level, maternal pre-pregnancy body mass index, parity, maternal smoking in pregnancy, and maternal fish intake in pregnancy. Abbreviations: As, inorganic arsenic; Cd, cadmium; Co, cobalt; CrI, credible interval; Cs, caesium; Cu, copper; DDE, dichlorodiphenyldichloroethylene; DDT, dichlorodiphenyltrichloroethane; HCB, hexachlorobenzene; Hg, mercury; MetS, Metabolic Syndrome; Mn, manganese; Mo, molybdenum; Pb, lead; PBDE, polybrominated diphenyl ether; PCBs, polychlorinated biphenyls; PFHxS, perfluorohexane sulfonate; PFNA, perfluorononanoic acid; PFOA, perfluoro-octanoic acid; PFOS, perfluoro-octane sulfonate; PFUnDA, perfluoroundecanoic acid.

© 2024 Güil-Oumrait N et al. *JAMA Network Open.*

eTable 18. Sensitivity Analyses. BWQS Models for Dichotomous MetS Risk (Low vs High) for All the Study Population

	MetS risk (n=1134)
**Exposure mixture group**	**OR (95%CrI)**
Metals	1.62 (1.40, 1.90)
OC pesticides	1.22 (1.13, 1.32)
PBDEs	1.08 (0.98, 1.20)
PFASs	1.11 (1.06, 1.14)
LMWPs	0.96 (0.90, 1.02)
HMWPs	1.05 (0.99, 1.10)

All models were adjusted for subcohort, parental country of birth, maternal age, maternal education level, maternal pre-pregnancy body mass index, parity, maternal smoking in pregnancy, and maternal fish intake in pregnancy. Abbreviations: BWQS, Bayesian weighted quantile sum; CrI, credible interval; HMWPs, high-molecular-weight phthalate metabolites; LMWPs, low-molecular-weight phthalate metabolites; MetS, Metabolic Syndrome; OC, organochlorine; OR, odds ratio; PBDEs, polybrominated diphenyl ethers; PFASs, perfluoroalkyl substances.

© 2024 Güil-Oumrait N et al. *JAMA Network Open.*

eReferences

1. Maitre L, De Bont J, Casas M, et al. Human Early Life Exposome (HELIX) study: A European population-based exposome cohort. *BMJ Open*. Published online 2018. doi:10.1136/bmjopen-2017-021311

2. Ahrens W, Moreno L, Mårild S, et al. Metabolic syndrome in young children: Definitions and results of the IDEFICS study. *Int J Obes*. Published online 2014. doi:10.1038/ijo.2014.130

3. Maitre L, Bustamante M, Hernández-Ferrer C, et al. Multi-omics signatures of the human early life exposome. *Nat Commun*. 2022;13(1):1-18. doi:10.1038/s41467-022-34422-2

4. Siskos AP, Jain P, Römisch-Margl W, et al. Interlaboratory Reproducibility of a Targeted Metabolomics Platform for Analysis of Human Serum and Plasma. *Anal Chem*. Published online 2017. doi:10.1021/acs.analchem.6b02930

5. Lau CHE, Siskos AP, Maitre L, et al. Determinants of the urinary and serum metabolome in children from six European populations. *BMC Med*. Published online 2018. doi:10.1186/s12916-018-1190-8

6. Nadarajah S, Kotz S. The exponentiated type distributions. *Acta Appl Math*. Published online 2006. doi:10.1007/s10440-006-9055-0

7. Colicino E, Pedretti NF, Busgang SA, Gennings C. Per- And poly-fluoroalkyl substances and bone mineral density: Results from the Bayesian weighted quantile sum regression. *Environ Epidemiol*. Published online 2020. doi:10.1097/EE9.0000000000000092

8. Rodushkin I, Axelsson MD. Application of double focusing sector field ICP-MS for multielemental characterization of human hair and nails. Part II. A study of the inhabitants of northern Sweden. *Sci Total Environ*. Published online 2000.

9. Ramon R, Murcia M, Aguinagalde X, et al. Prenatal mercury exposure in a multicenter cohort study in Spain. *Environ Int*. Published online 2011. doi:10.1016/j.envint.2010.12.004

10. Caspersen IH, Kvalem HE, Haugen M, et al. Determinants of plasma PCB, brominated flame retardants, and organochlorine pesticides in pregnant women and 3 year old children in The Norwegian Mother and Child Cohort Study. *Environ Res*. Published online 2016. doi:10.1016/j.envres.2015.12.020

11. Goñi F, López R, Etxeandia A, Millán E, Amiano P. High throughput method for the determination of organochlorine pesticides and polychlorinated biphenyls in human serum. *J Chromatogr B Anal Technol Biomed Life Sci*. Published online 2007. doi:10.1016/j.jchromb.2006.12.049

12. Koponen J, Rantakokko P, Airaksinen R, Kiviranta H. Determination of selected perfluorinated alkyl acids and persistent organic pollutants from a small volume human serum sample relevant for epidemiological studies. *J Chromatogr A*. Published online 2013. doi:10.1016/j.chroma.2013.07.064

13. Haug LS, Thomsen C, Becher G. A sensitive method for determination of a broad range of perfluorinated compounds in serum suitable for large-scale human biomonitoring. *J Chromatogr A*. Published online 2009. doi:10.1016/j.chroma.2008.10.113

14. Poothong S, Thomsen C, Padilla-Sanchez JA, Papadopoulou E, Haug LS. Distribution of Novel and Well-Known Poly- and Perfluoroalkyl Substances (PFASs) in Human Serum, Plasma, and Whole Blood. *Environ Sci Technol*. Published online 2017. doi:10.1021/acs.est.7b03299

15. Manzano-Salgado CB, Casas M, Lopez-Espinosa MJ, et al. Transfer of perfluoroalkyl substances from mother to fetus in a Spanish birth cohort. *Environ Res*. Published online 2015. doi:10.1016/j.envres.2015.07.020

16. Sabaredzovic A, Sakhi AK, Brantsæter AL, Thomsen C. Determination of 12 urinary

© 2024 Güil-Oumrait N et al. *JAMA Network Open.*

phthalate metabolites in Norwegian pregnant women by core-shell high performance liquid chromatography with on-line solid-phase extraction, column switching and tandem mass spectrometry. *J Chromatogr B Anal Technol Biomed Life Sci*. 2015;1002:343-352. doi:10.1016/J.JCHROMB.2015.08.040

17. Valvi D, Monfort N, Ventura R, et al. Variability and predictors of urinary phthalate metabolites in Spanish pregnant women. *Int J Hyg Environ Health*. 2015;218(2):220-231. doi:10.1016/J.IJHEH.2014.11.003

18. Sakhi AK, Sabaredzovic A, Papadopoulou E, Cequier E, Thomsen C. Levels, variability and determinants of environmental phenols in pairs of Norwegian mothers and children. *Environ Int*. 2018;114:242-251. doi:10.1016/J.ENVINT.2018.02.037

19. Philippat C, Mortamais M, Chevrier C, et al. Exposure to phthalates and phenols during pregnancy and offspring size at birth. *Environ Health Perspect*. Published online 2012. doi:10.1289/ehp.1103634

20. Cequier E, Sakhi AK, Haug LS, Thomsen C. Development of an ion-pair liquid chromatography–high resolution mass spectrometry method for determination of organophosphate pesticide metabolites in large-scale biomonitoring studies. *J Chromatogr A*. Published online 2016. doi:10.1016/j.chroma.2016.05.067

© 2024 Güil-Oumrait N et al. *JAMA Network Open.*
