# Supplementary material for: Prenatal Exposure to Chemical Mixtures and Metabolic Syndrome Risk in Children
Source: JAMA Netw Open. 2024 May 23;7(5):e2412040. doi: 10.1001/jamanetworkopen.2024.12040 (PMC11117089; doi:10.1001/jamanetworkopen.2024.12040)
Supplement: Supplement 2. — Data Sharing Statement [file jamanetwopen-e2412040-s002.pdf]

Data Sharing Statement

Güil-Oumrait. Prenatal Exposure to Chemical Mixtures and Metabolic Syndrome Risk in European Children. *JAMA Netw Open*. Published May 23, 2024.
doi:10.1001/jamanetworkopen.2024.12040

Data
Data available: No
